# Identification of the mRNA targets of tRNA-specific regulation using genome-wide simulation of translation

**DOI:** 10.1093/nar/gkw630

**Published:** 2016-07-12

**Authors:** Barbara Gorgoni, Luca Ciandrini, Matthew R. McFarland, M. Carmen Romano, Ian Stansfield

**Affiliations:** 1 University of Aberdeen, Institute of Medical Sciences, Foresterhill, Aberdeen AB25 2ZD, UK; 2 DIMNP - UMR 5235 & CNRS, Université de Montpellier, 34095 Montpellier, France; 3 Laboratoire Charles Coulomb UMR5221 & CNRS, Université de Montpellier, 34095 Montpellier, France; 4 University of Aberdeen, Institute for Complex Systems and Mathematical Biology, King's College, Aberdeen AB24 3UE, UK

## Abstract

tRNA gene copy number is a primary determinant of tRNA abundance and therefore the rate at which each tRNA delivers amino acids to the ribosome during translation. Low-abundance tRNAs decode rare codons slowly, but it is unclear which genes might be subject to tRNA-mediated regulation of expression. Here, those mRNA targets were identified via global simulation of translation. *In-silico* mRNA translation rates were compared for each mRNA in both wild-type and a ${\rm{tRNA}}_{{\rm{CUG}}}^{{\rm{Gln}}}$ *sup70-65* mutant, which exhibits a pseudohyphal growth phenotype and a 75% slower CAG codon translation rate. Of 4900 CAG-containing mRNAs, 300 showed significantly reduced *in silico* translation rates in a simulated tRNA mutant. Quantitative immunoassay confirmed that the reduced translation rates of sensitive mRNAs were ${\rm{tRNA}}_{{\rm{CUG}}}^{{\rm{Gln}}}$ concentration-dependent. Translation simulations showed that reduced ${\rm{tRNA}}_{{\rm{CUG}}}^{{\rm{Gln}}}$ concentrations triggered ribosome queues, which dissipated at reduced translation initiation rates. To validate this prediction experimentally, constitutive *gcn2* kinase mutants were used to reduce *in vivo* translation initiation rates. This repaired the relative translational rate defect of target mRNAs in the *sup70-65* background, and ameliorated *sup70-65* pseudohyphal growth phenotypes. We thus validate global simulation of translation as a new tool to identify mRNA targets of tRNA-specific gene regulation.

## INTRODUCTION

Translation of mRNA into protein represents the final stage of the gene expression pathway in which the transcribed mRNA is read by the ribosomal machinery, which translocates along the open reading frame to interpret the encoded peptide sequence. Its complexity can be likened to that of an industrial production line, involving not only the ribosomes and hundreds of ancillary translation factors, but also a population of transfer RNAs, of which there are 3 million in a yeast cell (1). In response to a cognate interaction between the tRNA anticodon and mRNA codon, tRNAs bring amino acids to the actively elongating ribosome at rates of up to 22 amino acids per second (2).

Due to genetic code redundancy, most amino acids are encoded by a family of codons, in turn recognised by more than one tRNA of a given amino acid-accepting type, the so called iso-acceptors. The different tRNAs of each iso-acceptor exhibit a particular cellular abundance dictated by that tRNA's gene copy number. In yeast, these vary by as much as 11-fold within a single isoacceptor class of tRNA (3). There is very good evidence that the concentration of tRNAs defines the rate of translation of its cognate codon(s), which can affect overall translational rate, but also protein folding and mRNA secondary structure interactions (4,5). For example, tRNA concentration controls the rate of translation elongation through a run of tandem codons of one type, and regulates the rate of translation of individual codons whose cognate tRNA is in low abundance (6–10). The frequency of ribosomal drop-off is also increased by the translational pause caused by a rare codon (11,12). tRNA concentration also regulates translational +1 frameshifting through control of the length of pause of the elongating ribosome (12–14). Most highly expressed genes, whose transcripts form a large proportion of the transcriptome (ribosomal protein mRNAs, glycolytic mRNAs) utilise codons that are translated by the most abundant tRNAs (15). This codon bias probably serves to avoid the detriment to cellular fitness caused by ribosome queuing in response to rare-tRNA induced ribosomal pausing.

Translation is the most resource- and energy-consuming process in the cell, and is therefore highly regulated by a range of protein *trans* factors in response to environment and nutrient availability (16–22). However, while it is clear that tRNA concentration can regulate the translation rate of individual codons, the extent to which translation of any given mRNA is regulated by tRNA concentration is unknown. Particularly unclear is the regulatory role played by low abundance codons; these are translated by a correspondingly rare tRNAs with known effects on translation pausing. Mathematical modelling of translation has been used to predict that globally, translation is governed principally by ribosome limitation (23–25), presumably ensuring that ribosomes are well-spaced on mRNAs. This can be described as initiation-regulated translation. This reflects the imperative that ribosome queuing incurs a fitness cost, and therefore that most mRNA translation should be initiation-regulated by ribosome availability. Indeed, when ribosome profiling was used to report the effects of depleting some types of yeast tRNA through gene deletion, no significant effects were seen on ribosome pausing, evidence used to argue for a minimal role for tRNA regulation of translation (26). Other studies however make the case that ribosome profiling does reveal pausing at non-optimal codons in yeast (8). Furthermore, a recent study using meticulous measurement of translation velocities on *Neurospora* mRNAs showed clearly that non-optimal codons significantly slow translation, while abundant codon stretches are rapidly translated (27). Supporting a regulatory role for rare codons, modelling of translation suggests that there are sub-populations of mRNAs whose translation are elongation-regulated (28,29), in which codons translated by low-abundance tRNAs play a regulatory role. These predictions were experimentally validated on artificial mRNAs by controlling the rate of initiation on reporters engineered to contain multiple rare codons (30). However, the extent to which rare codons can exert a regulatory influence on a wide range of natural mRNA sequences, and the requirements for rare codon disposition or configuration to achieve such regulation, is unclear.

A regulatory role for tRNAs in controlling translation is moreover strongly suggested by observations from a range of different organisms. In yeast, a genome-wide tRNA depletion initiative identified a wide range of growth phenotypes and transcriptional stress responses in yeast, particularly associated with deletion of tRNA genes with a low total gene complement (31). Also in yeast, mutant glutamine tRNAs cause the slowed decoding rate of tandem CAG codons (6), and confer a pseudohyphal growth phenotype (32). *Streptomyces bldA* mutants in the ${\rm{tRNA}}_{{\rm{UAA}}}^{{\rm{Leu}}}$ gene cause an inability to form aerial hyphae and produce antibiotics, because translation of mRNAs containing the extremely rare UUA codon is compromised (33,34). In *Escherichia coli*, mutations in a range of tRNAs specifically compromise phage lambda replication (35) or cause elevated mutagenesis frequencies (36), while in *Salmonella*, tRNA mutation of${\rm{\ tRNA}}_{{\rm{UCU}}}^{{\rm{Arg}}}$ specifically reduce production of fimbrae at the translational level (37). The levels of tRNA charging will also vary in response to amino acid starvation stress, and modelling predicts that in *E. coli* this may regulate groups of amino acid biosynthetic genes (38). More broadly, tRNA modification, which can affect tRNA stability and translational decoding properties, may be central to regulating particular genes, or groups of genes. Mutations in the yeast *TRM9* tRNA modification gene drive altered expression of genes enriched in codons targeted by Trm9-modified tRNAs (39). Similarly, mutations in the eukaryotic *Elongator* complex, catalysing a uridine tRNA wobble position modification, cause a range of specific phenotypic consequences including telomeric silencing, DNA damage responses, transcriptional elongation and exocytosis (40,41). This is likely to be due to altered translation of sub-sets of genes containing codon targets of the *Elongator*-modified tRNAs, since all *Elongator* phenotypes can be complemented by extra copies of key modified tRNAs (41).

Taken together, this body of evidence strongly suggests there is an important role played by the differential concentrations of tRNA species as regulators of the flux of ribosomes along each open reading frame, and thus, the gene-specific translational rate. To identify novel targets of gene expression regulation by low abundance tRNAs, we use global stochastic modelling of translation in *Saccharomyces cerevisiae* to predict the translational rate of every mRNA. Using this model we simulate the translational rate of each mRNA in a wild-type cell, versus that in a cell in which the concentration of a rare glutamine tRNA has been reduced 4-fold, mimicking the molecular phenotype of the yeast *sup70-65* allele of the ${\rm{\ tRNA}}_{{\rm{CUG}}}^{{\rm{Gln}}}$ (6). The mathematical model predicts several hundred genes that are sensitive to tRNA-specific regulation by this tRNA_Gln_, and we show using a focused experimental investigation that the model predictions are successfully validated. The use of global modelling of cellular translation, combined with experimental validation, reveals that although in global terms cellular translation is remarkably resilient to changes in tRNA concentration, nevertheless there are significant numbers of genes whose translational rate is sensitive to the concentrations of rare tRNAs. We show further that translational sensitivity to the concentration of any rare tRNA is determined not simply by the extent of use of the corresponding codon in an mRNA, but most likely by the configuration of those rare codons within the coding sequence, combined with the relative contents and dispositions of other types of rare codon in that gene. The configuration of rare codons, and their permutation with other rare codons, is thus revealed as an exquisitely sensitive modulator of gene expression.

## MATERIALS AND METHODS

### Mathematical modelling of translation

A stochastic model of translation was employed to simulate translation across yeast mRNAs (29). This model, based on the paradigmatic Totally Asymmetric Simple Exclusion Process (TASEP), represents the mRNA as a lattice, where each site of the lattice symbolises a codon (42,43). Ribosomes are then described as particles that hop onto the first site of the lattice, move along it translating the codons into amino acids, and hop off the lattice at the last site. Particles are considered to have a footprint of 9 codons to represent the actual ribosome width (44). Moreover, they cannot overtake each other, and a particle cannot initiate translation if the first 9 sites of the lattice are not free. Importantly, ribosomes advance through the lattice following a two-state dynamic: (1) recognition of the cognate tRNA with rate *k_i_* proportional to the concentration of that tRNA, and (2) translocation to the next codon with rate *γ* = 35 s^−1^, a rate independent of the specific codon (45). Thus our model simulates the stochastic movement of ribosomes along the mRNA, considering the actual ribosome width in terms of codons that they can cover, according to an exclusion process, i.e. two ribosomes cannot occupy the same codon. Importantly, the model also considers the internal biochemical cycle that the ribosome undergoes between each hopping event from one codon to the next. Hence, our model more closely represents the biomechanics of the translation process in comparison to other models (24,46,47) while preserving computational efficiency (each simulation only takes tenths of a second). mRNA-specific translation initiation rates α were derived using an integrated analysis of experimental data using model simulation as described previously (29). The termination rate β was considered not limiting and fixed equal to the fastest rate (i.e. *β* = *γ*) (48). In establishing the model, a series of simplifying assumptions were made; since there was no expectation that the depletion of a tRNA would affect ribosome biosynthesis, the ribosome concentration was invariant through the simulations. Likewise, tRNA charging by the aminoacyl tRNA synthetases was not expected to be affected, a decision made in part on the basis of experimental measurements of charging of the glutamine and histidine tRNAs in both wild-type and *sup70-65* mutant conditions (6), thus the proportions of tRNAs in the charged condition remained fixed. Likewise the ribosomal translocation rate (γ), following binding of the cognate tRNA, was invariant in the model.

This model predicts the average occupancy of each codon on the mRNA during translation, as well as the resulting translation rate, i.e. how many proteins per unit time are produced. Simulations were run by using a continuous time Monte-Carlo algorithm based on the Gillespie algorithm (49), coded in C++. Using this model, simulations for each of the 5500 yeast open reading frames (ORFs) were run until steady state was reached, after which then data were collected. Two sets of simulations were performed: (i) mRNA-specific translation initiation rates (designated α) were derived using an integrated analysis of experimental data using model simulation as described previously (29). Simulation of translation was carried out using a standard range of codon-specific decoding rates as described (29), or (ii) with the decoding rate of individual tRNAs (e.g ${\rm{tRNA}}_{{\rm{CUG}}}^{{\rm{Gln}}}$) decreased to 25% of their wild-type value to replicate a tRNA depletion condition. The translational rate *J* of any given mRNA, equivalent to a rate of synthesis of that protein, was recorded during the course of the simulation. Where required, *J* was recorded following simulation across a range of translation initiation values of α. The codon-dependent ribosomal density used to reconstruct the ribosome occupancy profile across the mRNA was extracted by identifying the codon position of the ribosomal A-site.

#### *S. cerevisiae* strains and growth conditions

Strains MLD17 (*MATa/α ade1/ade1 his3-11/ his3-1 1 trp1-1/ trp1-1 ura3-52/ ura3-52*) and MLD14 (*MATa/α sup70-65/sup70-65 ade1/ADE1 his3-11/his3-11 leu2-3,112/LEU2 trp1-1/trp1-1 ura3-52/ura3-52*) were kindly provided by Prof RA Singer (Dalhousie University, Halifax, Canada) (32). Cells were grown at 30^º^C on solid or liquid YPD (1% yeast extract, 2% peptone, 2% glucose) (50) or, after transformation, on the appropriate synthetic-defined (SD) or synthetic-complete (SC) selective medium (50). Where required, glucose was substituted with 1% galactose for gene induction.

#### Plasmids

Plasmids expressing HA-tagged open reading frames under the control of *GAL1* promoter (*FAR7*, YIL152W, *NDL1, STE18*, YDL012C, *ATG16, RCF1, LCL2, TRP4, ADH1, CDC19, MCM1, OPI1* and *PBP2*) were purchased from the Thermo Scientific Open Biosystems Yeast ORF Collection (Thermo Fisher Scientific Biosciences GmbH). To construct plasmids pYIL152-CAA, pFAR7-CAA and pNDL1-CAA, the respective open reading frames, in which all glutamine CAG codons were substituted with CAA codons, were synthesized by Eurofins Genomics and sub-cloned into the recipient plasmid BG1805 (51) by Gateway^®^ cloning (Life Technologies).

Over-expression of wild-type *SUP70* was driven by plasmid pSUP70-2μ (6). *GCN2* alleles cloned into yeast centromeric *URA3* vectors (wild-type; plasmid p722, E1522K; p915, E1537G; p914 and M719V-E1522K; p1055 (52)) were kindly provided by Prof G. Pavitt (University of Manchester, UK) and sub-cloned into pRS413 (53) following PCR amplification using primers AGGTCGACGGTATCGATTGTCCGATGAAGGTATGTAA and TAGAACTAGTGGATCCAAGCATTCT T CACGCCATAT (listed 5′-3′).

#### Western blot analysis

Whole cell protein extracts were prepared (54) from three independent yeast cultures grown in SC + 1% galactose medium until mid-log phase. Proteins were resolved on TGX stain-free pre-cast gels (Bio-Rad) and after transfer to a low-fluorescence PVDF membrane, protein content in each lane was quantified by UV epi-fluorescence and charge-coupled device camera to allow normalisation of protein loading. Protein expression of given HA-tagged yeast genes was determined using quantitative immuno-blot analysis, using an anti-HA primary antibody as specified by the manufacturer (HA.11 clone 16B12, Covance), and Super Signal West Femto chemi-luminescence kit (Thermo Scientific). Light output quantified using the charge-coupled device camera in an Alpha Innotech MultiImage II.

#### Northern blot analysis

Total RNA was extracted from cultures identical to those used for western blot analysis, using a Nucleospin RNA Extraction Kit (Macherey-Nagel). 5–10 μg of total RNA were used for northern blotting using the glyoxal denaturation method (55). A specific probe for the detection of HA-tagged overexpressed constructs was generated by PCR amplification of the BG1805 plasmid with primers GTGGTTGATGTGTCTAGAC and GTAAGATCTCATAGAACGCG (listed 5′-3′). For normalisation of loaded and transferred RNA samples, a specific probe for the yeast *SCR1* mRNA was amplified from genomic DNA using primers TCCTTCCTCGCGGCTAGA and CACCTTTGCTGACGCTGG (listed 5′-3′). PCR products were radio-labelled by random priming and RNA levels were quantified using a Fuji FLA-3000 phosphoimager.

#### Flow cytometry analysis

Three independent cultures of MLD14 and MLD17 strains (transformed with the p722 wild-type *GCN2* or M719V/E1522K *gcn2^C^* allele plasmid p1055 as described above) were grown in 5ml YPD media to mid-log phase. One millitre samples of each culture were harvested, washed and resuspended in sterile phosphate buffered saline. The forward-scatter (FSC) of samples was measured using the blue 488 nm laser in a Becton Dickinson LSR Fortessa Cell Analyser. Typically 20 000 cells were analysed for each sample and the same cytometer settings (photomultiplier tube values, etc) were used throughout the experiment. The resulting data was analysed using FlowJo (version 10). To analyse the data, a population gate was created for the control culture containing the 95% lowest FSC value population. By applying this gate to cultures or strains representing the test population, the percentage of cells with FSC larger than 95% of the wild-type population was determined to define pseudohyphal cell chains.

#### Plasmid retention assay

Cytometry analysis was carried out on cells grown in non-selective YPD medium to optimise chain formation. The extent of plasmid retention was quantified during growth on non-plasmid selective media for the *gcn2^C^-*encoding plasmids that conferred a growth disadvantage. Three independent cultures of yeast strains MLD14 (*sup70-65/sup70-65*) and MLD17 (*SUP70* wild-type; transformed with wild-type p722 or plasmid p1055 expressing the M719V/E1522K *GCN2* allele as described above) were grown to mid-log phase in YPD media. Cells were harvested and plated for single colonies on either complete medium to quantify total colony-forming units, or on selective medium to identify plasmid transformants. The percentage retention of *GCN2* allele plasmids could thus be calculated. Plasmid retention in strain MLD17 was typically 59% for wild-type *GCN2* transformants and 22% for plasmids carrying the constitutively active *gcn2*-M719V-E1522K allele.

## RESULTS

### Cell-wide modelling of translation identifies the targets of tRNA regulation of translation

**Figure 1.**
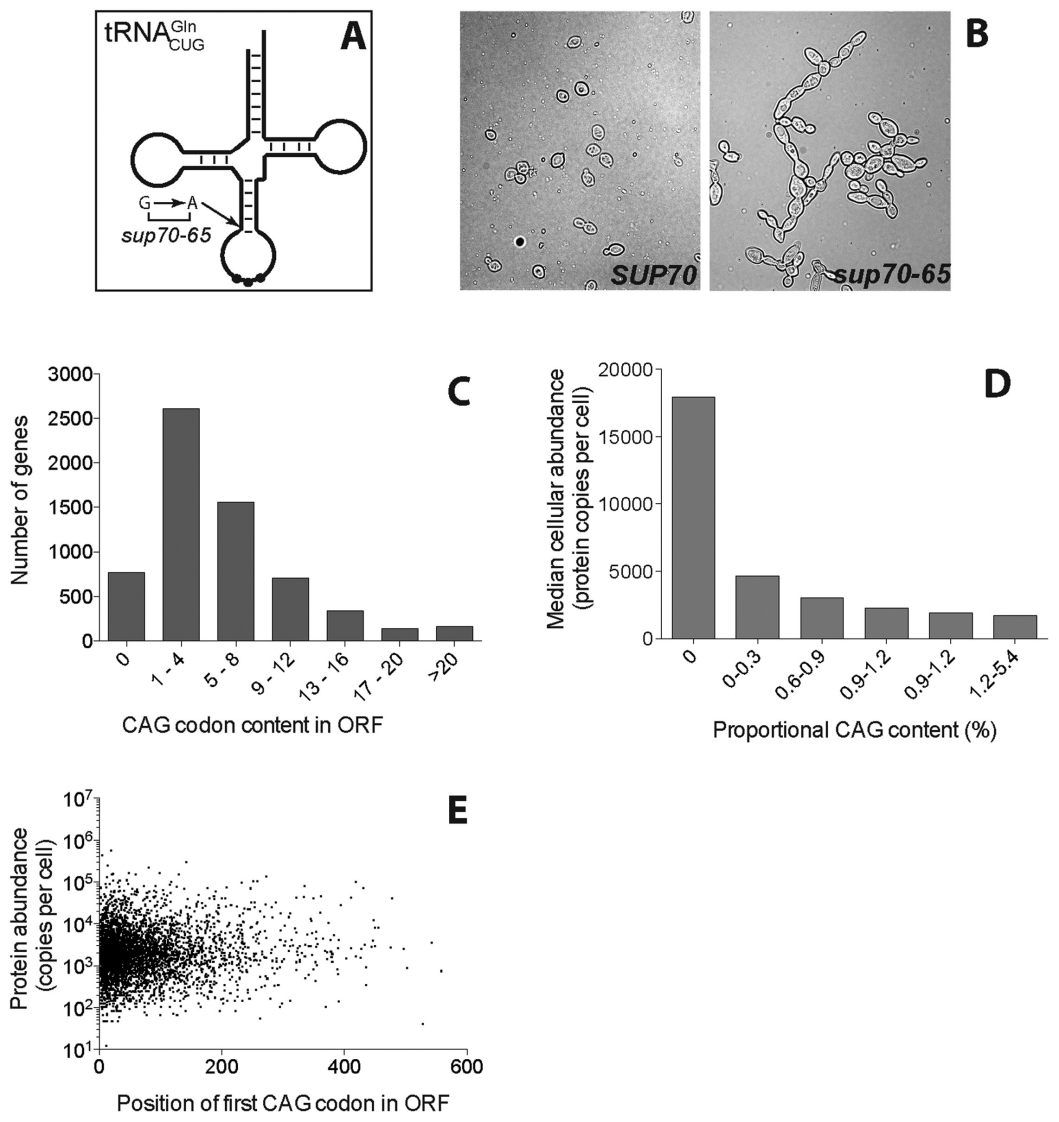
CAG codon usage in the transcriptome. (**A**) The CAG glutamine codon is recognised by the single copy *SUP70* gene, encoding ${\rm{tRNA}}_{{\rm{CUG}}}^{{\rm{Gln}}}$. The *sup70-65* allele defines a variant lacking a base-pairing interaction at the base of the anticodon stem. (**B**) The *sup70-65* mutation causes a pseudohyphal growth phenotype. (**C**) The CAG frequencies were recorded for all *S. cerevisiae* ORFs. (**D**) The cellular abundance of yeast proteins (66) was plotted against the binned proportional content of CAG codons relative to ORF length. (**E**) For each yeast ORF, the position of the first (5′-most) CAG codon was recorded and plotted against the cellular protein abundance.

There are known examples where a tRNA can regulate the expression of specific sets of genes, e.g. the *Streptomyces bldA* tRNA that controls sporulation and antibiotic production (34). Mutant alleles of the *Saccharomyces cerevisiae SUP70* gene encoding ${\rm{tRNA}}_{{\rm{CUG}}}^{{\rm{Gln}}}$ exhibit a slowed translation rate of the cognate CAG codon caused by a 4-fold reduced abundance of ${\rm{tRNA}}_{{\rm{CUG}}}^{{\rm{Gln}}}$ (6) (Figure 1A). This in turn triggers inappropriate nitrogen-starvation responses in N-replete growth media, including the constitutive formation of pseudohyphal chains of cells in this normally single-celled fungus (32) (Figure 1B). However it is unclear if this reduction in the abundance of an already rare tRNA affects translation rates globally, or if translation of specific sub-sets of mRNAs are particularly prone to alterations in ${\rm{tRNA}}_{{\rm{CUG}}}^{{\rm{Gln}}}$ abundance. If the latter, this group of transcripts must encode a protein(s) required for suppression of the pseudohyphal response in a wild-type cell. The identification challenge is considerable; for example, over 2500 yeast genes have between 1 and 4 CAG codons, and only 600 ORFs are CAG-free (Figure 1C). As a simple consequence of codon bias, highly expressed proteins are CAG-free. However, for CAG-containing mRNAs there is a very weak correlation between the cellular abundance of a protein and its mRNA's CAG content (Figure 1D). Therefore it is likely that the absolute content of CAG codons is not a determining factor in gene expression, but rather the position of CAG codons in the ORF (56). Slowly translated CAG codons situated early (5′) in an ORF may cause queuing of ribosomes back to the mRNA cap, inhibiting efficient ribosome recruitment. However, there is no correlation between position of the first CAG codon, and protein abundance (Figure 1E). It was therefore not possible to use bioinformatic approaches alone to predict which mRNAs might be specifically targeted due to inefficient decoding by ${\rm{tRNA}}_{{\rm{CUG}}}^{{\rm{Gln}}}$.

**Figure 2.**
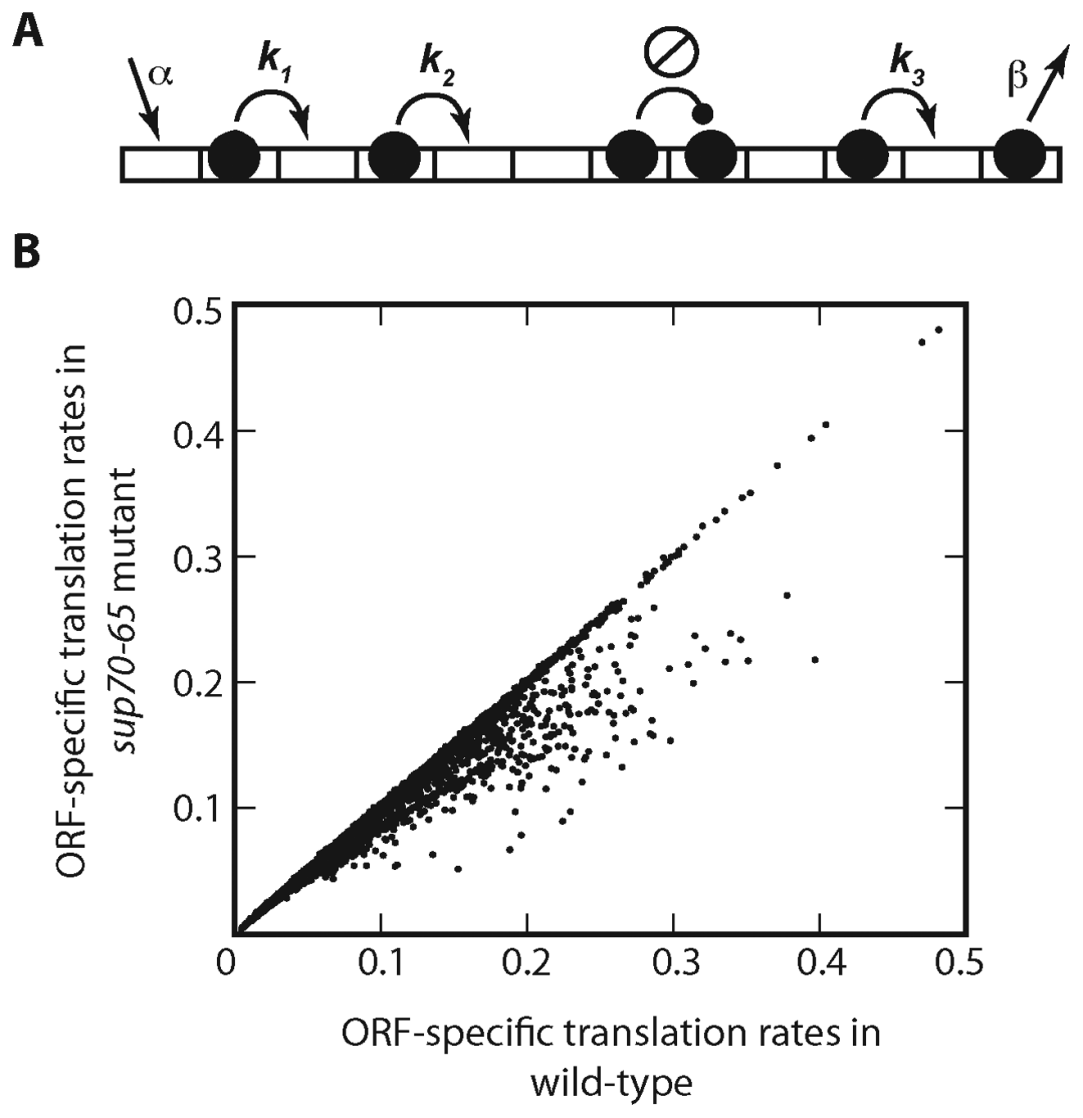
Modelling ribosome flux on the total yeast transcriptome identifies the targets of glutamine ${\rm{tRNA}}_{{\rm{CUG}}}^{{\rm{Gln}}}$ regulation. (**A**) Mathematical modelling of ribosome flux along each mRNA employed a Totally Asymmetric Simple Exclusion Process (TASEP) in which ribosomal particles of 9-codon width join a lattice representing the mRNA with rate α, and translate the lattice, codon by codon using 2-state dynamics. These are defined by cognate tRNA finding rate *k_i_*, dictated by tRNA abundance, and translocation rate γ for ribosomes charged with the cognate tRNA, not shown in the figure (67). Ribosome then terminate with rate *β*. (**B**) The TASEP model was used to simulate in turn each of the 5500 yeast mRNAs using published initiation and stepping rates (29). Simulations were performed using wild-type tRNA concentrations, and again using a concentration of ${\rm{tRNA}}_{{\rm{CUG}}}^{{\rm{Gln}}}$ reduced to 25% of normal levels to mimic the *sup70-65* mutant as determined experimentally (6). Translation rates for each mRNA under the two conditions are plotted.

We instead adopted a novel alternative approach to identify the targets of ${\rm{tRNA}}_{{\rm{CUG}}}^{{\rm{Gln}}}$ regulation, and simulated the translation of all 5500 yeast open reading frames (ORF), using a two-state ribosome model of translation (Materials and Methods; Figure 2A). The rate of translation of each ORF was simulated in the first instance using wild-type yeast tRNA concentrations. Simulations were then repeated using the tRNA complement of a *sup70-65* mutant, in which the concentration of ${\rm{tRNA}}_{{\rm{CUG}}}^{{\rm{Gln}}}$ was reduced to 25% of the wild-type value, as determined experimentally (6).

Comparison of the rates of translation of each ORF in wild-type and tRNA mutant backgrounds revealed that although the majority of mRNAs were unaffected by the reduction in CAG-decoding tRNA, nevertheless there were approximately 300 target mRNAs that the model simulation predicted would be as much 2-fold down-regulated (Figure 2B). The translation of these ORFs clearly responds markedly to reductions in the level of the single copy yeast ${\rm{tRNA}}_{{\rm{CUG}}}^{{\rm{Gln}}}$, identifying these genes as potential targets for regulation by a single-copy tRNA.

### Specific mRNAs are regulated by rare-tRNA abundance

The model predictions (Figure 2B), generated using our mathematical model of translation, were then validated experimentally. Proteins whose translational expression was predicted to be compromised by the reduction in concentrations of ${\rm{tRNA}}_{{\rm{CUG}}}^{{\rm{Gln}}}$ in the yeast *sup70-65* mutant were quantified in wild-type and *sup70-65* backgrounds.

Accordingly, a range of 8 ORFs was selected whose expression was predicted by the simulation to be sensitive to ${\rm{tRNA}}_{{\rm{CUG}}}^{{\rm{Gln}}}$ levels. One example in this group was *FAR7*, an ORF of 221 codons containing 12 CAG codons. A control group was also selected, comprising ORFs whose reading frames in some cases contained significant numbers of CAG codons, but whose translation was nevertheless predicted by the model simulation to be insensitive to levels of the ${\rm{tRNA}}_{{\rm{CUG}}}^{{\rm{Gln}}}$. For example, *MCM1* contains 29 CAG codons in an ORF of length 286, but was nevertheless predicted to be unresponsive to ${\rm{tRNA}}_{{\rm{CUG}}}^{{\rm{Gln}}}$. CAG contents for all genes are listed in Supplementary Table S1.

**Figure 3.**
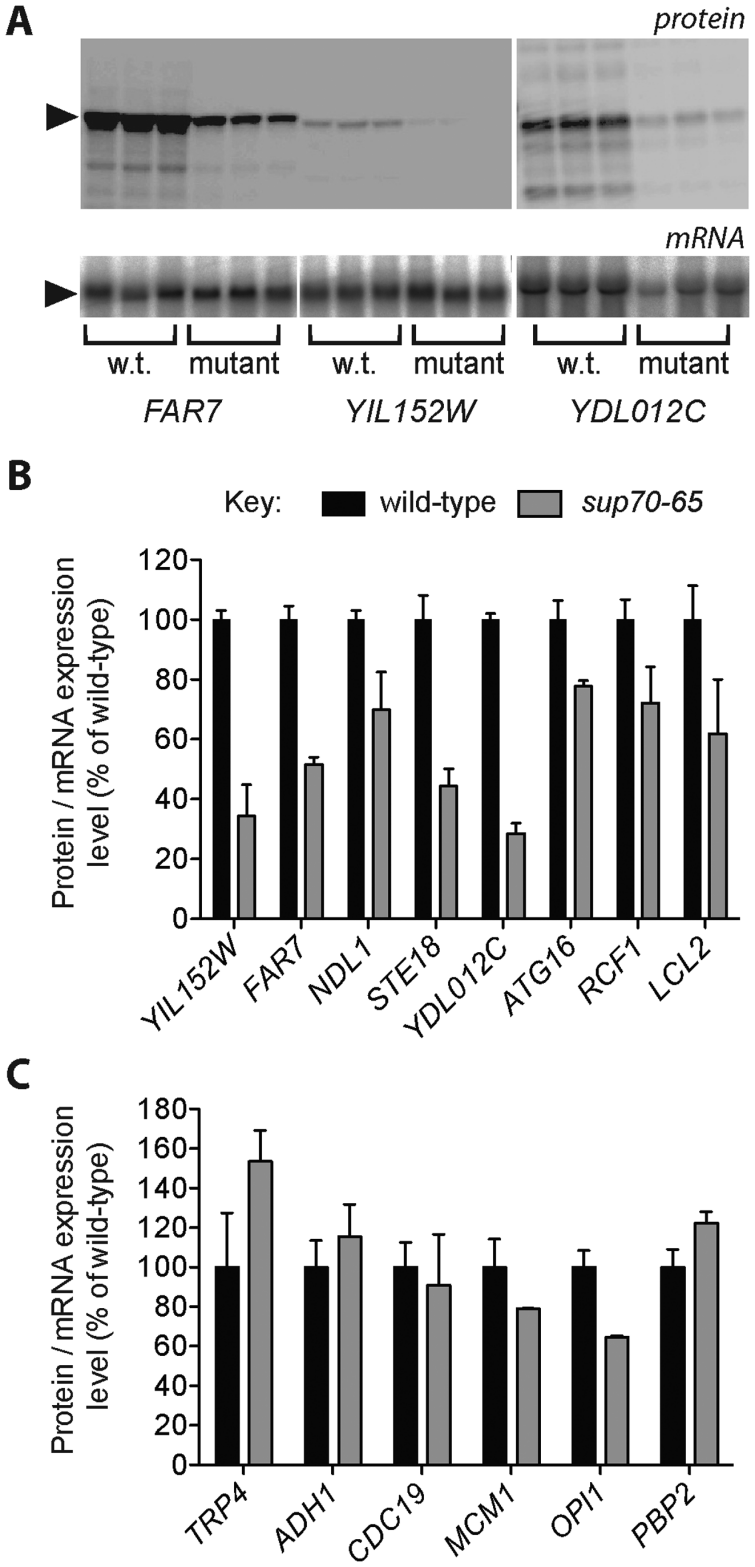
Experimental confirmation of the targets of glutamine ${\rm{tRNA}}_{{\rm{CUG}}}^{{\rm{Gln}}}$ regulation. (**A**) Quantitative Western blotting was performed for target proteins (predicted to be sensitive to cellular levels of ${\rm{tRNA}}_{{\rm{CUG}}}^{{\rm{Gln}}}$) HA-tagged and expressed in either wild-type or *sup70-65* mutant yeast; three examples are shown. Triplicate biological replicates were assayed for HA-tagged protein expression following gel loading normalisation. RNA expression levels for each HA-tagged construct were quantified using Northern blots, normalised for loading using an *SCR1* control probe. (**B**) *CAG-regulated mRNAs;* HA-tagged protein expression levels were quantified in wild-type (black bars) and *sup70-65* yeast (grey bars) for eight putative targets of ${\rm{tRNA}}_{{\rm{CUG}}}^{{\rm{Gln}}}$ regulation (*n* = 3, ± standard error of the mean). Protein expression levels were normalised using the levels of the corresponding mRNA expression levels determined by northern blot. (**C**) *Non-CAG-regulated mRNAs;* The same process was repeated for a series of control ORFs, whose expression is not predicted to be responsive to ${\rm{tRNA}}_{{\rm{CUG}}}^{{\rm{Gln}}}$ (*n* = 3, ± standard error of the mean).

These ORFs, tagged with an HA epitope-protein A fusion, were expressed in both wild-type and *sup70-65* mutant yeast under control of the *GAL* (galactose-regulatable) promoter on a plasmid. The transformants were grown exponentially, then total protein and RNA was isolated for analysis by quantitative Western and Northern blot from three independent biological replicates, normalised for gel loading in each case (Figure 3A). Northern blot phosphoimager data was used to quantitate mRNA levels, which were used to normalise mean protein expression levels of each of the target ORFs. Any effects of alterations in transcription or mRNA stability were thus excluded.

This analysis was conducted both for the test ORF set predicted to be ${\rm{tRNA}}_{{\rm{CUG}}}^{{\rm{Gln}}}$–sensitive, and for the control ${\rm{tRNA}}_{{\rm{CUG}}}^{{\rm{Gln}}}$-insensitive gene set (Figure 3B and C respectively). The results clearly show that the expression levels of the tRNA_CUG_-sensitive ORFs are significantly reduced across the range of genes tested (a mean of 54% relative to the wild-type control; Figure 3B *cf* model predictions, Supplementary Table S1) while the control set of tRNA_CUG_-insensitive ORFs, selected because model simulation identified their translational rate as unaffected by levels of the glutamine tRNA, showed a mean expression level in the *sup70-65* mutant of 103% relative to wild-type. The measured reductions in protein expression caused by the *sup70-65* mutation (Figure 3B) were highly correlated with the original model simulation quantitative predictions of reduced translation (Figure 2 and Supplementary Table S1), with a correlation coefficient *R*^2^ = 0.63.

Taken together, the results showed clearly there are genes in yeast that are sensitive to variations in the levels of rare tRNAs, and that *in silico* simulation of translation can successfully predict the identity of those mRNA sequences. Moreover, the experimental validation of the model predictions for control ORFs demonstrates that alone, the content of a rare codon such as CAG in an ORF is not predictive of its sensitivity to the concentration of its cognate tRNA.

### Specific translation defects in the *sup70-65* mutant are ${\rm{tRNA}}_{{\rm{CUG}}}^{{\rm{Gln}}}$-dependent

**Figure 4.**
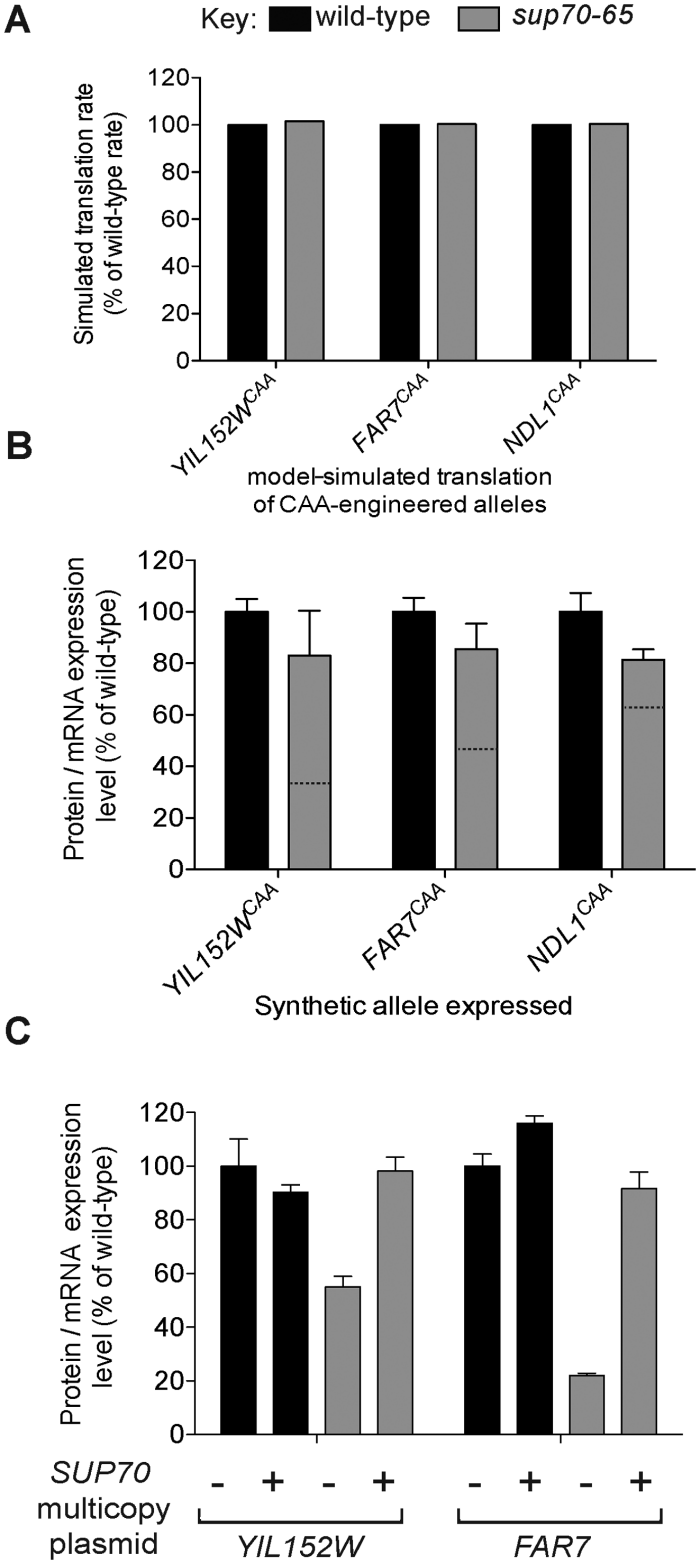
Codon engineering of genes regulated by ${\rm{tRNA}}_{{\rm{CUG}}}^{{\rm{Gln}}}$ ablates their sensitivity to rare glutamine codon concentration. (**A**) Three target mRNAs known to be under-expressed in a *sup70-65* yeast were selected. *In silico*, all CAG codons within each ORF were replaced by synonymous CAA codons. Using the two-state TASEP model, their translation efficiency was compared in a wild-type and *sup70-65* simulation (dark and light grey bars respectively). (**B**) Synthetic variants of the same three ORFs were prepared (e.g. *FAR7^CAA^*) in which all CAG codons were replaced by CAA. Their expression levels were compared in wild-type (dark bars) and *sup70-65* mutant yeast (light bars) using quantitative Western blotting normalised for mRNA expression level as in Figure 3 (*n* = 3, ± SE). For reference, the expression level of the progenitor, CAG-containing allele, (Figure 3B data) is shown by the dashed lines. (**C**) Expression levels of ${\rm{tRNA}}_{{\rm{CUG}}}^{{\rm{Gln}}}$-sensitive target genes YIL152W and *FAR7* were compared in wild-type and *sup70-65* mutant yeast using quantitative Western blotting normalised by measured mRNA levels, in the presence and absence of ectopically-expressed *SUP70* wild-type ${\rm{tRNA}}_{{\rm{CUG}}}^{{\rm{Gln}}}$ (*n* = 3, ± SE). The tRNA-encoding *SUP70* gene was transformed on a multicopy plasmid to ensure high-level ${\rm{tRNA}}_{{\rm{CUG}}}^{{\rm{Gln}}}$ expression.

In order to confirm that the compromised translational efficiency measured (Figure 3) was due to the presence of CAG codons in the open reading frame, the mathematical model of translation was first used to predict the effect of replacing all CAG codons with the synonymous CAA glutamine codon. As expected, for the three candidate CAG-sensitive genes chosen, replacement of all CAG codons with CAA rendered the *in silico* translation immune to a simulated reduction of ${\rm{tRNA}}_{{\rm{CUG}}}^{{\rm{Gln}}}$ concentrations (Figure 4A).

The model predictions were then confirmed experimentally. Alleles of the YIL152W, *FAR7*, and *NDL1* genes were synthesised in which all CAG codons were replaced by their synonymous CAA counterpart. Expression of these CAA-replacement alleles was assessed using quantitative Western blots, normalised for mRNA concentration as before. The results showed clearly that the expression levels of each of the genes in the *sup70-65* mutants was restored to almost wild-type levels, identifying the presence of the CAG codons as the sole cause of the reduced expression in the *sup70-65* mutant (Figure 4B, compare with Figure 3B).

To further confirm the mechanism via which translational efficiency (translational rate) of these three genes is compromised in the ${\rm{tRNA}}_{{\rm{CUG}}}^{{\rm{Gln}}}$ mutant, both the wild-type and the *sup70-65* mutant were separately transformed with a multi-copy plasmid expressing a wild-type copy of the *SUP70* gene, encoding ${\rm{tRNA}}_{{\rm{CUG}}}^{{\rm{Gln}}}$. This is known to complement the *sup70-65* pseudohyphal growth phenotype, and normalise the CAG codon translation rate (32). As predicted, the ectopic tRNA expression in the mutant restored the translational efficiency of both of the two tested mRNAs to wild-type levels (Figure 4C).

### The relative rates of translation initiation and elongation govern the sensitivity of mRNA translation to rare-tRNA concentrations

Whereas wild-type yeast grows in single budded cell form, a yeast *sup70-*65 mutant forms long, pseudohyphal chains of cells. We show that the reduced ${\rm{tRNA}}_{{\rm{CUG}}}^{{\rm{Gln}}}$ concentrations in the *sup70-65* mutant cause reduced expression of a specific sub-set of CAG-containing genes (Figure 3), and we suggest that it is this failure to translate one or more specific mRNAs at wild-type rates that triggers the formation of pseudohyphae. However, how reduced concentrations of ${\rm{tRNA}}_{{\rm{CUG}}}^{{\rm{Gln}}}$ inhibit the translational rate of some mRNAs is unclear. Reducing the concentration of a given tRNA stochastically reduces the translation rate of its cognate codon (6–10). Such extended translational pauses can cause ribosomal queuing, which if they extend back to the 5′ end of the mRNA can compromise recruitment of ribosomal subunits to the 5′ mRNA cap, and thus translational efficiency of the mRNA (30). We therefore hypothesised that the reduced translation rate of CAG codons produces ribosome queues which inhibit ribosome recruitment on the YIL152W and *FAR7* mRNAs, explaining why they exhibit reduced expression in the *sup70-65* mutant.

**Figure 5.**
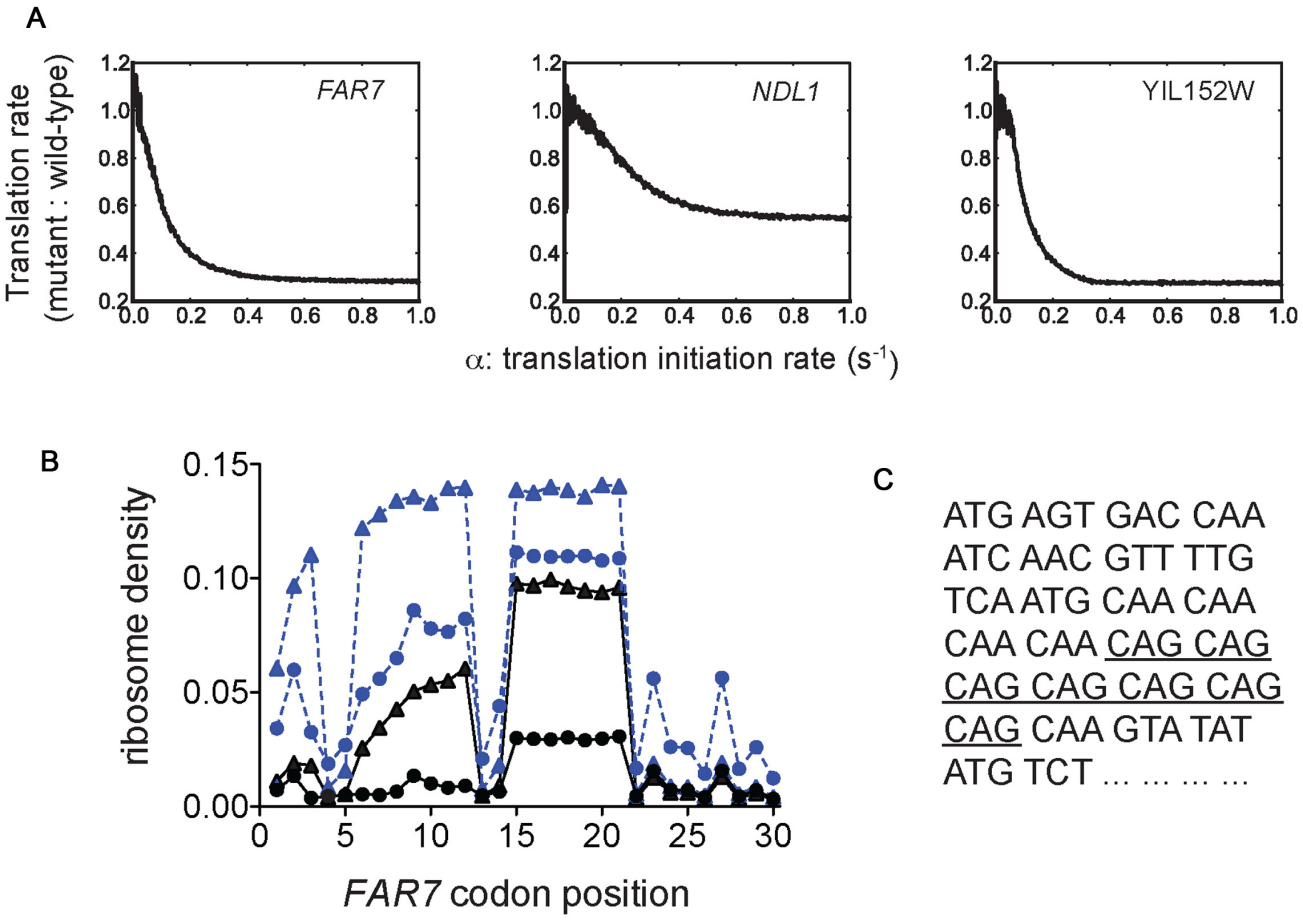
The rate of translation elongation at CAG codons, relative to the rate of translation initiation, governs ribosome queue formation and thus ${\rm{tRNA}}_{{\rm{Gln}}}^{{\rm{CUG}}}$ sensitivity. (**A**) TASEP simulation of translation of three ${\rm{tRNA}}_{{\rm{CUG}}}^{{\rm{Gln}}}$-sensitive mRNAs was conducted across a range of values of α, the translation initiation rate (Figure 2). Simulations were conducted in either a wild-type tRNA background, or a *sup70-65* tRNA background, and a ratio of these translational efficiencies plotted against each value of the translation initiation rate. (**B**) These same TASEP simulations were used to record the codon-specific ribosomal density across the *FAR7* ORF to indicate the positions of ribosome queuing. The ribosomal density across the *FAR7* was recorded in a wild-type strain (filled circle symbols) and the *sup70-65* mutant condition (open triangle symbols), at the physiological initiation rate of 0.3 events/s (dashed lines, blue symbols), and again at a 6-fold slower rate of 0.05 events/s (solid lines). The ribosomal density across codons 1–30 is presented, showing that the ribosomal density at the mRNA 5′ end in the *sup70-*65 mutant is greater than that in the wild-type at the high initiation rate, but that ribosome queues dissipate at the lower initiation rate, eliminating this density differential at the 5′-most codons. (**C**) Codons 1–24 of the *FAR7* open reading frame, with the positions of the CAG codons indicated (underlined).

If the translation rate is being limited by a rate-limiting step at the elongation stage, forming a ribosomal queue, then significantly reducing the rate of translation initiation will introduce a more rate-limiting step earlier in the translation process, at the point of ribosome joining to the mRNA. This in turn will cause the ribosomal queue to dissipate. Using the mathematical model of translation we confirmed that this is in fact the case by simulating translation of three genes known to be sensitive to tRNA_CUG_ concentrations; for each gene the ratio of translation rate in *sup70-65* to that in wild-type tends to a value of 1 as the *in silico* translation initiation rate is decreased. This signifies that the *sup70-65* translational defect, relative to wild-type, should be masked at low rates of translational initiation (Figure 5A).

The model simulation was then used to quantitate the ribosome density profile across the *FAR7* mRNA. In the simulated *sup70-65* background, ribosome densities at the 5′ end of the *FAR7* mRNA were significantly raised at high initiation rates (Figure 5B), indicative of ribosome queuing. In contrast, when the *in silico* initiation rate was reduced, ribosome queues were reduced, and ribosomal density at the earliest, 5′-most, codon in the ORF was reduced to wild-type levels in the simulated ${\rm{tRNA}}_{{\rm{CUG}}}^{{\rm{Gln}}}$ mutant condition. To confirm the queuing behaviour was specific for the predicted ${\rm{tRNA}}_{{\rm{CUG}}}^{{\rm{Gln}}}$-sensitive mRNAs such as *FAR7*, we also simulated translation on the CAG-rich, but ${\rm{tRNA}}_{{\rm{CUG}}}^{{\rm{Gln}}}$-insensitive *MCM1* mRNA. As expected, at the physiological initiation rate, ribosome densities at the *MCM1* 5′ end were identical in wild-type and *sup70-65* simulations (Supplementary Figure S1), explaining why Mcm1 protein expression levels in the *sup70-65* mutant were indistinguishable from that in the wild-type (Figure 3C). The model prediction was thus clear; the mutant ${\rm{tRNA}}_{{\rm{CUG}}}^{{\rm{Gln}}}$ induces the formation of ribosome queues on ${\rm{tRNA}}_{{\rm{CUG}}}^{{\rm{Gln}}}$-sensitive mRNAs such as *FAR7*, queues which should dissipate when the translation initiation rate in the *sup70-65* background is reduced, thus masking the *sup70-65* mutant phenotypes.

To experimentally validate this model prediction, we employed constitutively-active mutants of the Gcn2 protein kinase (*gcn2^C^*) to reduce the global rate of translation initiation *via* phosphorylation of the essential translation initiation factor eIF2-α (52). We reasoned that reducing the translation initiation rate would make translation initiation, rather than translation elongation, the rate-limiting step in translation, and prevent the formation of ribosome queues at CAG codons that would otherwise extend to the mRNA 5′ end. This would eliminate the translational disadvantage suffered by these mRNAs in a *sup70-65* translation system, relative to their expression level in a wild-type cell. Ameliorating the translational block should in turn diminish the severity of the *sup70-65* mutant phenotypes, including pseudohyphal chain formation. We therefore transformed a plasmid bearing a *gcn2^C^* allele into either wild-type or *sup70-65* yeast, and used cell cytometry and, separately, direct counting of cell chains, to quantify the effect on the pseudohyphal growth phenotype.

**Figure 6.**
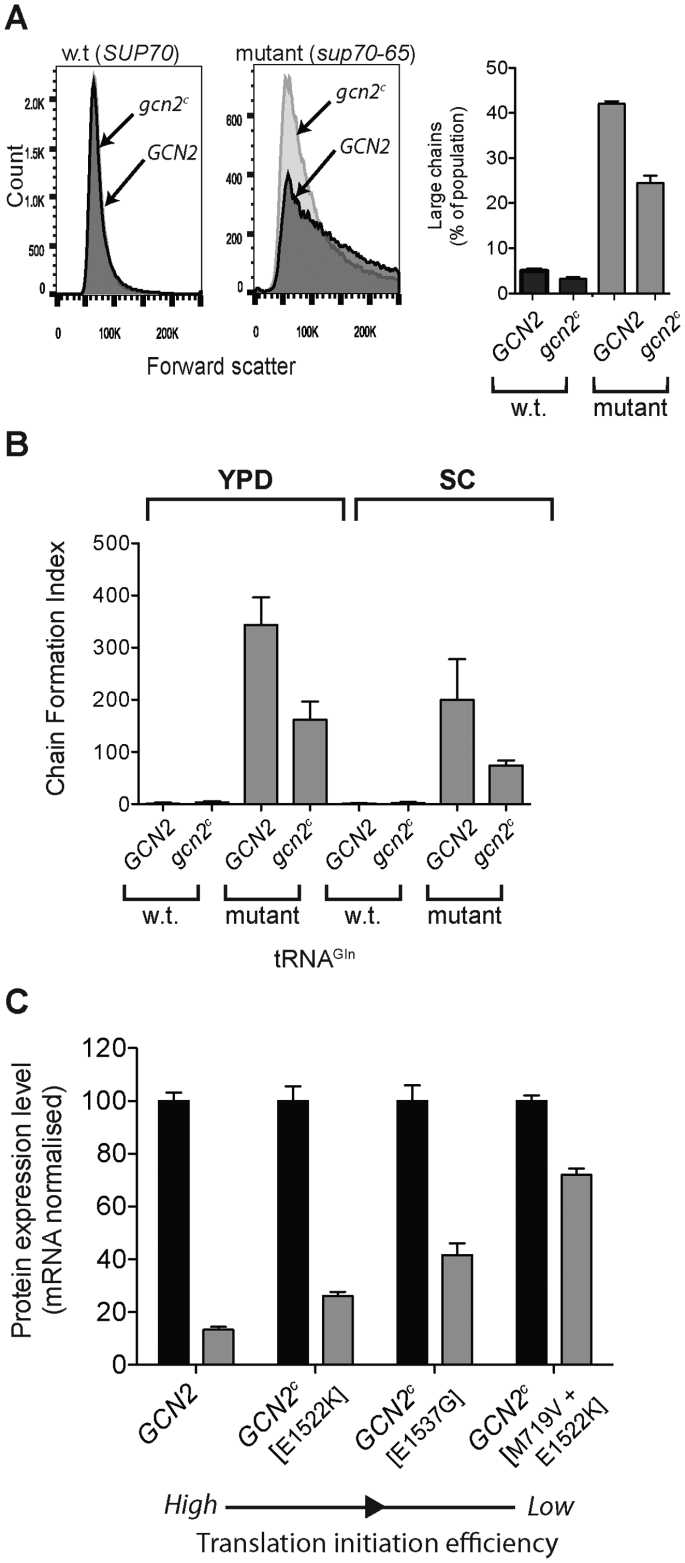
Reductions in the global translation initiation rate ameliorate the mRNA-specific translational inefficiencies caused by reduced ${\rm{tRNA}}_{{\rm{CUG}}}^{{\rm{Gln}}}$ concentration. (**A**) Cell cytometry was used to assay the prevalence of pseudohyphal chains that typify the *sup70-65* mutant. Forward scatter measurements indicated the extent of formation of large chains of cells. Pseudohyphal growth was assessed in wild-type and *sup70-65* yeast in the presence of either a wild-type ectopic *GCN2* allele (dark-shaded frequency plot), or a constitutively-active *gcn2^c^* allele (light-shaded). Population sizes of large chains and single budded cells were quantified using the cytometry data and plotted in the bar chart. (**B**) To confirm these observations, the degree of pseudohyphal chains formation in wild-type or *sup70-65* cells, transformed with a plasmid expressing either *CGN2* or *gcn2^c^* was assessed by direct microscope observation. Pseudohyphal chains were counted, and a chain formation index used to capture the extent of pseudohyphal growth during growth on complete (YPD) or minimal medium (SC) (6). (**C**) HA-tagged *FAR7* was expressed in wild-type and *sup70-65* yeast transformed with ectopically expressed *CGN2* or *gcn2^c^* genes to either maintain, or reduce, global translation initiation rates respectively. Three different *gcn2^c^* alleles were used with increasing degrees of constitutive eIF2 phosphorylation activity (E1522K < E1573G < M719V, E1522K). *FAR7* expression was quantified using Western blotting, normalised for mRNA expression level as in Figure 3 (*n* = 3, ± standard error of the mean). The Far7p expression level in the mutant *sup70-65* was expressed as a percentage of the expression level in a wild-type cell.

Cytometric analysis, using forward scatter as an indicator of cell size, showed clearly that whereas the wild-type population exists as single cells, a significant proportion of a *sup70-65* population is composed of cell chains exhibiting large forward scatter (Figure 6A). Crucially, reducing the translation initiation rate through expression of the *gcn2^C^* allele in the *sup70-65* mutant caused a significant, almost 2-fold shift in the population away from chains and towards single cells (Figure 6A, bar chart). That change may have even been greater had there not been a significant loss of the growth-inhibitory *gcn2^C^* expressing plasmid from the transformed cells (Materials and Methods; 22%-59% plasmid retention), caused by the requirement to grow the cells under plasmid non-selective conditions.

Direct microscopic observation of cells allowed quantification of a chain formation index to indicate the extent of pseudohyphal formation in wild-type and *sup70-65* strains (6). This revealed that *gcn2^C^* expression caused at least 2-fold reductions in chain formation in the *sup70-65* mutant (Figure 6B). Thus the cell developmental phenotype caused by reductions in rare tRNA concentration can be significantly reduced through down regulation of the global translation initiation rate.

It was important to verify that a *gcn2^C^*-driven reduction in the translation initiation rate can also improve the impaired translation of a *sup70-65*-sensitive mRNA such as *FAR7*, as predicted by the *in silico* translation simulation (Figure 5A). A number of *gcn2^C^* constitutive alleles have been identified, exhibiting a range of eIF2-α kinase activities and thus slowed growth phenotypes (52). These were employed to produce a range of translation initiation rates. In separate experiments, three different *gcn2^C^* alleles exhibiting low, medium or high constitutive eIF2 kinase activities were transformed into either wild-type or *sup70-65* strains, and the translation of *FAR7* mRNA monitored, normalised relative to their mRNA levels.

The results revealed that expressing constitutively active Gcn2 protein in the *sup70-65* strain increased the translational efficiency of *FAR7* mRNA relative to that in wild-type cells (Figure 6C). Moreover, a graded, increasing response of *FAR7* mRNA translational efficiency was recorded in the *sup70-65* background in response to expressing *gcn2^C^* alleles of increasing constitutive activity; E1562K, E1537G or M719V/E1522K. Note that as the constitutive eIF2 phosphorylation activity increases across this series, so the translation initiation rate decreases. We also observed that the measured content of Far7p relative to total cell protein, (i.e. prior to ‘percentage of wild-type’ normalisation, Figure 6C) increased in the mutant background as the initiation rate decreased. Although the translation rate of Far7p in the mutant background is expected to decrease with decreasing initiation rate, it does so at a slower rate than in the wild type background, and also slower than the average protein in the cell. Thus as the initiation rate is reduced, Far7p content as a fraction of total cellular protein increases in the mutant background because the absence of ribosome queues at low initiation rates allows the *FAR7* mRNA to more effectively recruit ribosomes. The physiological ratio of translation efficiency of *FAR7* mRNA relative to the translation efficiency of the mRNA cellular pool is thus restored. Together, this analysis indicated that reducing translation initiation rates caused an amelioration of the elongation-inhibitory effects of slow codons within an mRNA.

### Low abundance tRNAs can act as master regulators of specific subsets of mRNAs

The demonstration that the rare glutamine ${\rm{tRNA}}_{{\rm{CUG}}}^{{\rm{Gln}}}$ is able to regulate a specific set of mRNA translation events raised the possibility that other tRNAs might exhibit similar regulatory potential to govern the translation rate of specific sub-sets of mRNAs. For example, the abundance of yeast ${\rm{tRNA}}_{{\rm{CCU}}}^{{\rm{Arg}}}$ is known to be low enough to trigger a ribosomal pause-driven ribosomal frameshift in certain contexts (12). The potential exists for it also to regulate translation elongation directly through ribosome stalling events, causing ribosome queuing.

**Figure 7.**
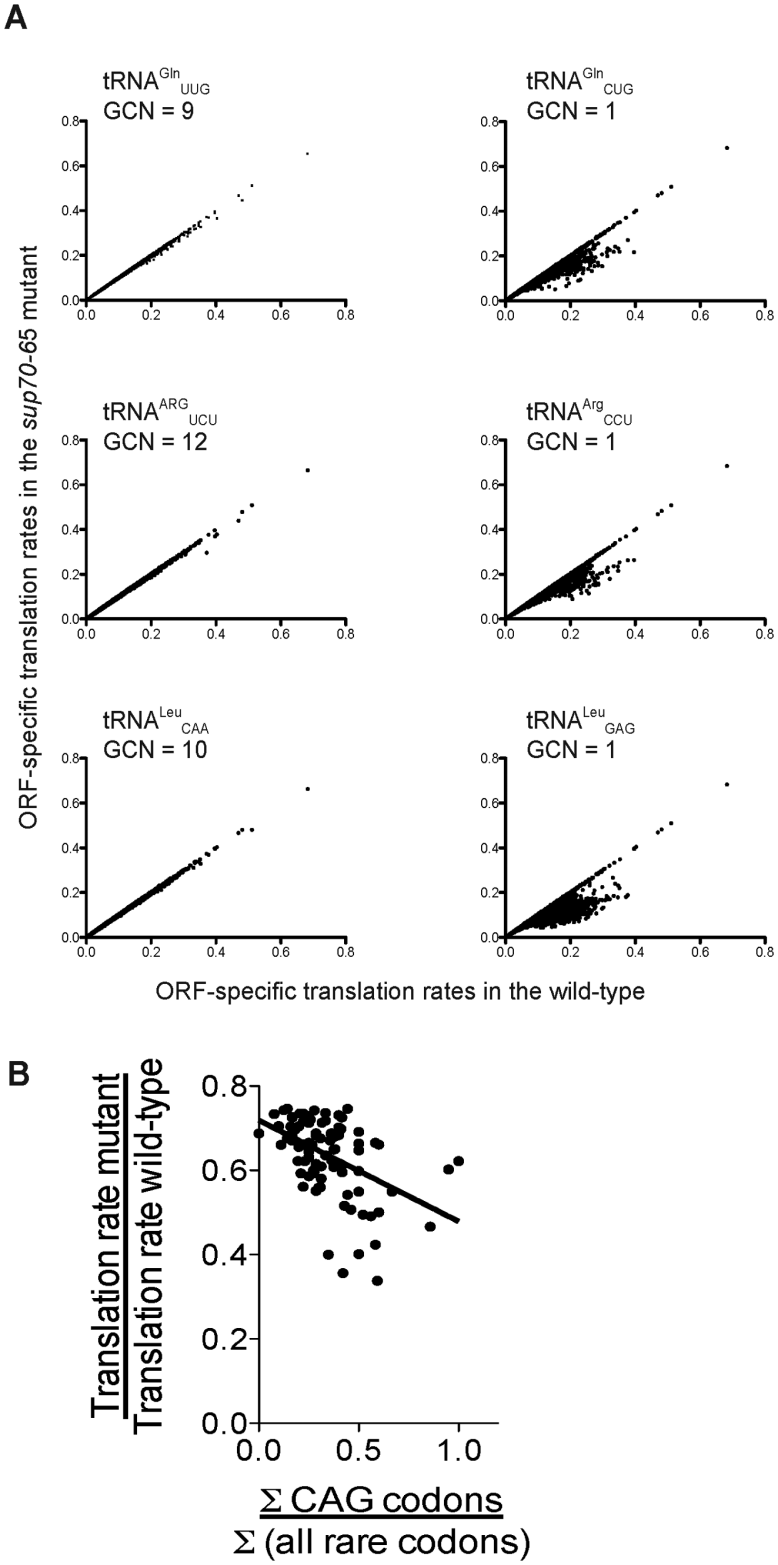
Translation is sensitive to changes in concentrations of rare tRNAs. (**A**) Translation of all 5500 yeast ORFs was simulated using the TASEP model representing a wild-type spectrum of tRNA concentrations, and again for a simulated cell where the concentration of one tRNA type was reduced to 25% of wild-type levels. The wild-type translation rate for each ORF is plotted against the corresponding tRNA-depleted rate. This exercise was repeated for all 42 species of yeast cytoplasmic tRNA. The results for 6 tRNAs are presented, three types whose encoding genes are multicopy (Gene Copy Number GCN > 4; left column), and three single gene-copy tRNAs (GCN = 1; right column). (**B**) A disproportionately high content of CAG codons within an ORF, relative to other rare codons, together with the configuration of those CAG codons, may drive ORF sensitivity to ${\rm{tRNA}}_{{\rm{CUG}}}^{{\rm{Gln}}}$ concentrations. To test this, for the 90 most *sup70-*65 sensitive ORFs, the CAG rare codon content, normalised to content of other rare codons, was plotted against the translation efficiency ratio (*sup70-65*/wild-type) revealing a negative correlation (*R*^2^ = 0.21).

In order to survey the potential for all the yeast tRNAs to regulate translation at the elongation stage via ribosome queuing, translation of all *circa* 5500 yeast mRNAs was simulated under 42 different conditions; in each, the concentration of a different species of tRNA was reduced to 25% of its wild-type level. In each case, the resulting mRNA translational efficiencies were compared with those in cells with a wild-type tRNA population as described earlier for ${\rm{tRNA}}_{{\rm{CUG}}}^{{\rm{Gln}}}$ (Figure 2B). The results showed clearly that the rare tRNAs, encoded by single copy tRNA genes, could all exert a regulatory effect on specific groups of yeast mRNAs. The translational rate of between 300 and 700 mRNAs was reduced when the concentration of either ${\rm{tRNA}}_{{\rm{CUG}}}^{{\rm{Gln}}}$, or any other single copy tRNA such as ${\rm{tRNA}}_{{\rm{CCU}}}^{{\rm{Arg}}}$ and ${\rm{tRNA}}_{{\rm{GGA}}}^{{\rm{Leu}}}$, was reduced to 25% of its normal concentration in the simulated translation system (Figure 7A, Supplementary Table S2).

Importantly, a similar, simulated reduction in the concentration of abundant tRNAs (in this case defined as those with a gene copy number > 4) produced no effect on translational rate of any mRNA. This indicated that translation is extremely robust to marked changes in the concentrations of abundant tRNAs.

Each of the sets of genes showing sensitivity to the concentration of a given rare tRNA did not overlap with one another, indicating that codon content and configuration render given mRNAs sensitive to particular rare tRNA species. Thus some coding sequences may contain disproportionate numbers of a rare codon of a given type configured in a particular arrangement so as to trigger queue formation, making that mRNA the target for regulation by alterations in the concentration of its cognate tRNA. To test this hypothesis, we examined whether the sensitivity of a given mRNA to reductions in the concentration of ${\rm{tRNA}}_{{\rm{CUG}}}^{{\rm{Gln}}}$ was inversely correlated with a specific content of CAG codons, combined with the absence of other rare codons (ratio of CAG codons: other rare codons). Indeed a weak negative correlation was observed (Figure 7B), indicating that ORFs containing populations of rare codons of type *i*, but lacking significant numbers of other rare codon types *j,k,l* may exhibit sensitivity to the concentration of *i* codon-cognate tRNA. Of course, this correlation analysis omits information on codon configuration and displacement, probably explaining why the correlation coefficient is relatively low. Overall the analysis reveals that low abundance tRNAs, in combination with the codon content and configuration of the open reading frame, can play a fundamental role in regulating gene expression at the translational level.

## DISCUSSION

The role of tRNA abundance in controlling the efficiency of mRNA translation has been the subject of much debate, with some research concluding that control of translation initiation, rather than elongation, is by far the most dominant effect on translational efficiency (26,57), or that ribosome limitation is key to controlling translation efficiency (23). The importance of these influences cannot be overstated, nevertheless there is mounting evidence that tRNAs can and do regulate sub-sets of mRNAs (34). mRNAs may therefore fall into two classes, those that are principally initiation-regulated, and those that are elongation-regulated and responsive to tRNA concentration (28–30). The challenge then is to identify which mRNAs are sensitive to the concentration of any given tRNA. Only then will it be possible to understand how dynamic changes in tRNA concentration can control expression of some genes at the translational level.

In this work, we investigated an unusual mutant form of an essential, single gene copy tRNA in yeast that reduces the translation rate of CAG glutamine codons by 75% while still maintaining viability (6,32). This molecular phenotype causes a constitutive pseudohyphal growth phenotype (Figure 1), almost certainly through tRNA-driven changes in gene expression in the *sup70-*65 mutant. The *sup70-65* mutant thus represents a powerful tool to analyse the effects of depleting an essential tRNA species. However, identifying the mRNA translation events sensitive to this tRNA required a new approach. For the first time we used global simulation of translation across all 5500 yeast mRNAs to replicate either wild-type translation, or translation in a *sup70-65* mutant background. In this way, we effectively carried out an *in silico* genetic screen, identifying the putative targets of translation regulation by the ${\rm{tRNA}}_{{\rm{CUG}}}^{{\rm{Gln}}}$ (Figure 2B) and experimentally validating these predictions using quantitative immunoblot (Figure 3). Importantly, we were able to prove that the translation defect was dependent on both the presence of CAG codons and the concentration of ${\rm{tRNA}}_{{\rm{CUG}}}^{{\rm{Gln}}}$ (Figure 4). Moreover, we identified the mechanism responsible for the reduced rate of translation of these mRNAs; slowed CAG translation generated ribosome queues, which the evidence indicates inhibited efficient ribosome recruitment to the mRNA 5′ end. We further showed that this ribosome queuing could be dissipated by a reduction in global translation initiation rates, leading to a restoration of translation efficiency (Figures 5 and 6). The engineered reduction in translation initiation rates as expected caused slowed growth of the yeast, and presumably reductions in the pool of available ribosomes and tRNAs. However, these effects apply equally to the tRNA mutant and wild-type strains, with the wild-type measurements controlling for the effect of reduced growth on the expression of the target mRNAs.

This analysis thus identifies a group of mRNAs whose translational rate is specifically reduced in the *sup70-65* background. It is assumed that the failure to efficiently translate one or more of the proteins they encode underpins the pseudohyphal growth phenotype of the mutant. We therefore examined the list of genes identified by the *in silico* screen for candidates known to regulate pseudohyphal differentiation (58). Indeed, some genes with a known link to pseudohyphal growth were also targets of regulation by ${\rm{tRNA}}_{{\rm{CUG}}}^{{\rm{Gln}}}$ (e.g. *HAP2, ERV25, NDL1*; Supplementary Table S1). At this stage however, it is unclear whether the pseudohyphal growth phenotype has a simple monogenic basis, or as likely, is a composite phenotype caused by simultaneously reducing the expression level of a number of proteins. A broader question is whether expression of groups of *S. cerevisiae* genes with a shared function are controlled by single-copy tRNA abundance. However, inspection of those target genes predicted to be underexpressed as a result of single copy tRNA depletion did not reveal significant over-representation of any gene ontologies (data not shown). This does not however exclude the possibility that key transcription factors might be regulated through rare tRNA abundance, with pleiotropic effects.

The amino acid glutamine sits at the nexus of a large number of nitrogen regulatory cell circuits, and it has been suggested the glutamine tRNAs themselves may act as sensing or regulatory molecules in some way, since the *sup70-65* morphological phenotype indicates a deregulated response to N-starvation (32). However, with any tRNA-driven phenotype, the most obvious mechanism for a phenotypic effect must be via translation itself. In that context, a thorough investigation of nitrogen starvation responses in yeast has revealed that the *sup70-*65 mutation prevents the normal sequestration of the Gln3 transcription factor in the nucleus (59). Gat1 re-location to the nucleus is also blocked under some N-starvation signalling conditions (59). Following a series of detailed N-starvation time-course experiments that take advantage of *sup70-65* temperature sensitivity, the authors concluded that the most likely way in which *sup70-65* mutant tRNA would block Gln3 dynamics is if a component were needed for Gln3 nuclear relocalisation that ‘possessed an exquisitely specific and concentration sensitive codon bias for glutamine tRNA_CUG_ (59). We concur with their prediction, and in this work identify 300 proteins whose translation is indeed uniquely sensitive to CAG translation due to the configuration of CAG codons within their encoding mRNA. Identification of Gln3 regulatory and trafficking proteins within our sensitive mRNA target group can now be addressed.

Our work has identified the translation regulatory targets of a key single copy tRNA when that tRNA is significantly depleted through mutation. This prompts the obvious question of how tRNA concentrations might vary physiologically in a wild-type cell to control gene expression at the translational level. The view that concentrations of individual tRNA species are fixed, and determined only by tRNA gene copy number is looking increasingly simplistic. We know that the developmental regulation of the *Streptomyces bldA* tRNA is not a special case (60), since as long ago as 1994 researchers were reporting differential regulation of the four members of the *S. cerevisiae* seryl-tRNA family in response to growth rate and carbon source (61). Differential, albeit moderate, regulation of the *E. coli* tRNAs in response to growth rate has also been reported (62). More recently, tRNA abundance in human was reported to vary across tissue type (63,64), and in *Lactococcus* sp in response to growth conditions (65). Intriguingly, in *Lactococcus*, it is the concentration of low abundance tRNAs that primarily responds to growth rate, exactly the sub-population that our study predicts has the greatest regulatory role on mRNA translation (Figure 7).

Our study also reveals the regulatory potential inherent in the selection of rare codon types in each ORF. Simulated depletion of each of the single copy tRNAs in yeast revealed in each case several hundred genes that were translationally down-regulated in response to depletion of a single gene copy tRNA (Figure 7). These gene sets were however largely non-overlapping (Supplementary Table S2). Thus evolutionary selection of particular rare codon types within an ORF, and the potential exclusion of other rare codon types could render an ORF sensitive to one particular rare tRNA species. Indeed, there was some evidence that examples of this might exist; we showed that mRNAs most sensitive to ${\rm{tRNA}}_{{\rm{CUG}}}^{{\rm{Gln}}}$ depletion contained a high ratio of CAG codons relative to other non-CAG rare codons (Figure 7B). This could offer a partial explanation for why some CAG-containing mRNAs were sensitive to ${\rm{tRNA}}_{{\rm{CUG}}}^{{\rm{Gln}}}$ concentrations, and other ORFs with a high CAG content were insensitive (Figure 3). However, the configuration of those CAG codons is also of paramount importance; many CAG-containing genes are completely unaffected by the *sup70-65* tRNA milieu, and we show that in one such insensitive gene, *MCM1*, containing 25 CAG codons, the ribosomal density at the 5′ end is not significantly altered in the *sup70-65* background relative to wild-type, underlining the importance of configuration.

This study has unequivocally clarified our understanding of the regulatory role that can be played by low abundance tRNAs. The translational pausing that results when a ribosome translates a rare tRNA's cognate codon can cause a bottleneck in translation elongation. If this rate-limiting step is slower than the rate of translation initiation on that mRNA, ribosome queues will then result. Ribosome queues that extend back to the 5′ cap of an mRNA, representing an elongation ‘bottleneck’, will lead to reduced translational efficiency through failure to compete for, and recruit, new ribosomal subunits to the now occluded 5′ cap. We thus show the crucial role in determining translational efficiency played by the balance between initiation and elongation rates. In addition to this fundamental insight into the control of gene expression by tRNAs, our study has also broken new ground through the use of an *in silico* screen to rapidly probe transcriptome-wide translational rate in a computer model. This approach opens the door for completely new analytical approaches to understanding translational regulation on a system-wide scale in the future.

## Supplementary Material

SUPPLEMENTARY DATA
