# Supplementary material for: Identification of the mRNA targets of tRNA-specific regulation using genome-wide simulation of translation
Source: Nucleic Acids Res. 2016 Jul 12;44(19):9231–44. doi: 10.1093/nar/gkw630 (PMC5100601; doi:10.1093/nar/gkw630)
Supplement: SUPPLEMENTARY DATA [file supp_44_19_9231__index.html]

Identification of the mRNA targets of tRNA-specific regulation using genome-wide simulation of translation — SUPPLEMENTARY DATA 

# Identification of the mRNA targets of tRNA-specific regulation using genome-wide simulation of translation

## SUPPLEMENTARY DATA

- SUPPLEMENTARY DATA
- SUPPLEMENTARY DATA
- SUPPLEMENTARY DATA
- SUPPLEMENTARY DATA
