# Supplementary material for: Identification of the mRNA targets of tRNA-specific regulation using genome-wide simulation of translation
Source: Nucleic Acids Res. 2016 Jul 12;44(19):9231–44. doi: 10.1093/nar/gkw630 (PMC5100601; doi:10.1093/nar/gkw630)
Supplement: SUPPLEMENTARY DATA [file supp_gkw630_nar-01103-n-2016-File009.docx]

**Figure S1:** The *MCM1* CAG-rich mRNA is translation initiation, not elongation-limited in a *sup70-*65 tRNA mutant

[A] Model simulation of translation of a non-$\text{tRNA}_{\text{CUG}}^{\text{Gln}}$-sensitive mRNA, *MCM1* was carried out at the physiological value of α=0.09, the translation initiation rate (Fig 2). Simulations were conducted to represent either a wild-type tRNA background, or a *sup70-65* tRNA background.

These model simulations were used to record the codon-specific ribosomal density across the *MCM1* ORF to indicate the positions of ribosome queuing. The ribosomal density across *MCM1* was recorded in a wild-type strain (filled circle symbols) and the *sup70-65* mutant condition (filled blue triangle symbols), at the physiological initiation rate of 0.09 events/s (dashed lines, blue symbols). The ribosomal density across codons 1-100 is presented, showing that the ribosomal density at the mRNA 5' end across the first 15 codons measured in wild-type and mutant *sup70-65* tRNA conditions is approximately equal. There is thus no ribosome density differential on the *MCM1* mRNA between *sup70-65* mutant and wild-type at the 5'-most codons, explaining why the translational efficiency of *MCM1* mRNA is not significantly different between wild-type and *sup70-65* cells (Figure 3).

[B] Codons 1-120 of the *MCM1* open reading frame, with the positions of the CAG codons indicated (underlined, bold).
