# Supplementary material for: Identification of the mRNA targets of tRNA-specific regulation using genome-wide simulation of translation
Source: Nucleic Acids Res. 2016 Jul 12;44(19):9231–44. doi: 10.1093/nar/gkw630 (PMC5100601; doi:10.1093/nar/gkw630)
Supplement: SUPPLEMENTARY DATA [file supp_gkw630_nar-01103-n-2016-File010.pdf]

Figure S2: Simulated translation of all yeast mRNAs in wild-type tRNA concentrations, and with the concentration of each of the yeast tRNA types depleted by 75% in turn. This approach yielded 41 sets of transcriptome-wide simulations.

Each graph illustrates a scatter plot of the translation rate *J* of each mRNA in the two conditions, derived using the stochastic model of translation as described (Materials and Methods). Where the points lie along the x=y axis, translation of those mRNAs is unaffected by depletion of the tRNA named in that graph's title. Where points lie to the right of the x=y axis, the rate of translation *J* of that mRNA has been reduced in the tRNA depletion condition, when compared to translation in a wild-type tRNA milieu.

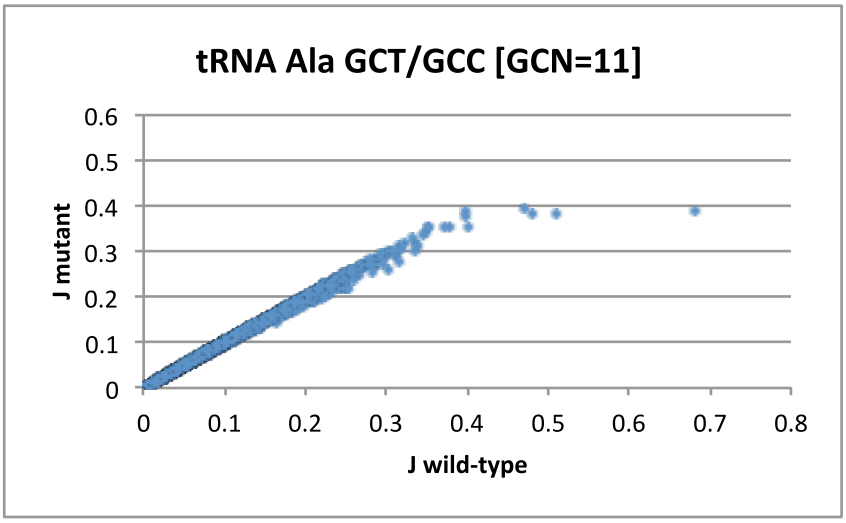

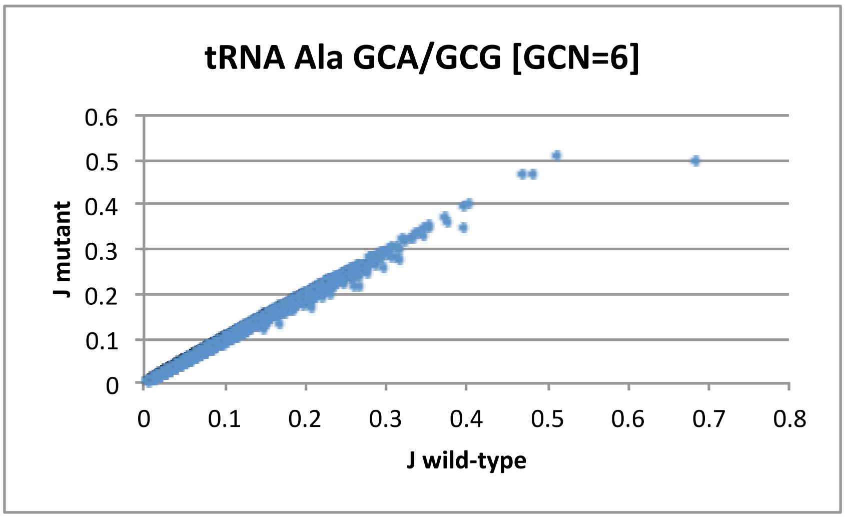

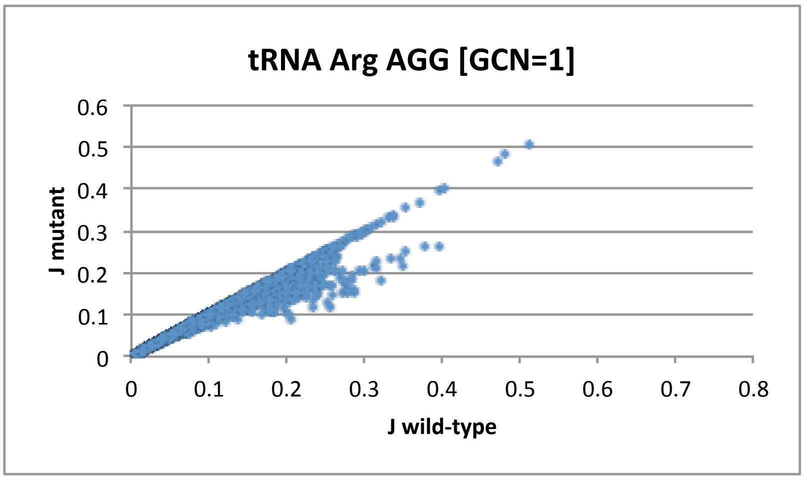
tRNA Arg AGG [GCN=1]
0.6
0.5
0.4
0.3
0.2
0.1
0
J mutant
0
0.1
0.2
0.3
0.4
0.5
0.6
0.7
0.8
J wild-type

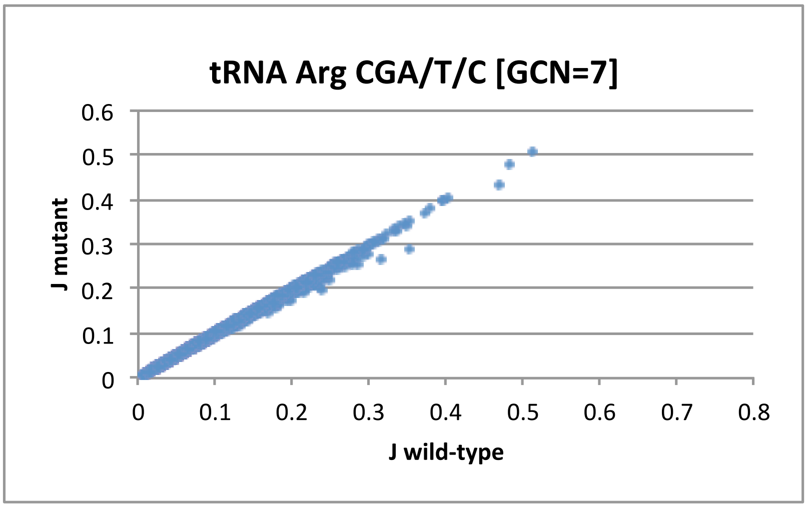
tRNA Arg CGA/T/C [GCN=7]
0.6
0.5
0.4
0.3
0.2
0.1
0
J mutant
0
0.1
0.2
0.3
0.4
0.5
0.6
0.7
0.8
J wild-type

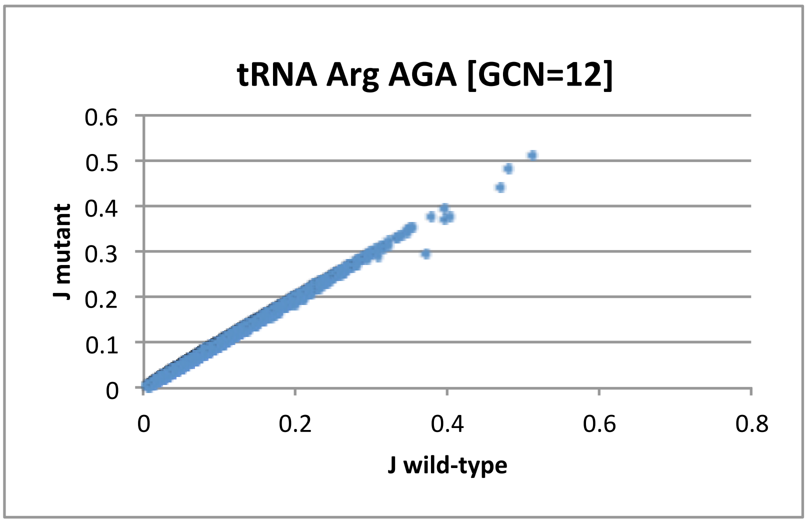
tRNA Arg AGA [GCN=12]
0.6
0.5
0.4
0.3
0.2
0.1
0
J mutant
0
0.2
0.4
0.6
0.8
J wild-type

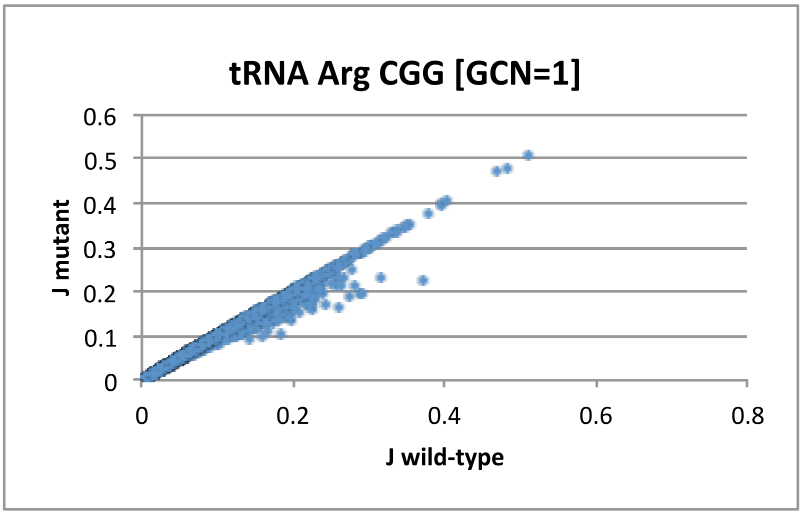
tRNA Arg CGG [GCN=1]
J mutant
0
0.1
0.2
0.3
0.4
0.5
0.6
0
0.2
0.4
0.6
0.8
J wild-type

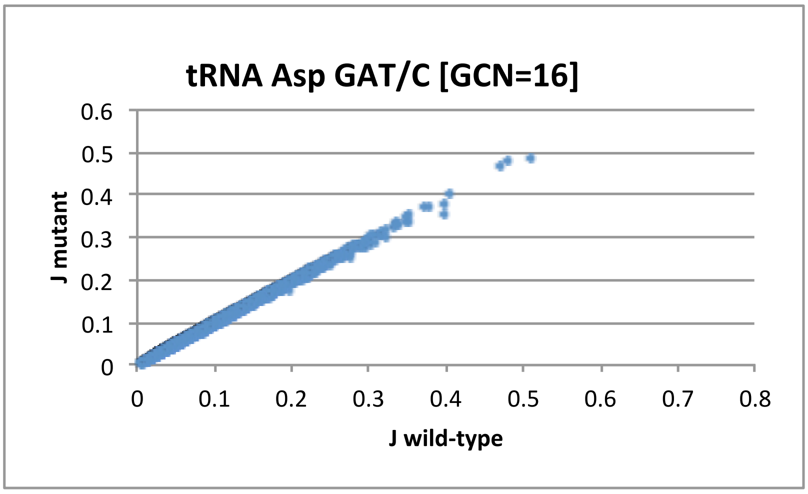
tRNA Asp GAT/C [GCN=16]
J mutant
0
0.1
0.2
0.3
0.4
0.5
0.6
0
0.1
0.2
0.3
0.4
0.5
0.6
0.7
0.8
J wild-type

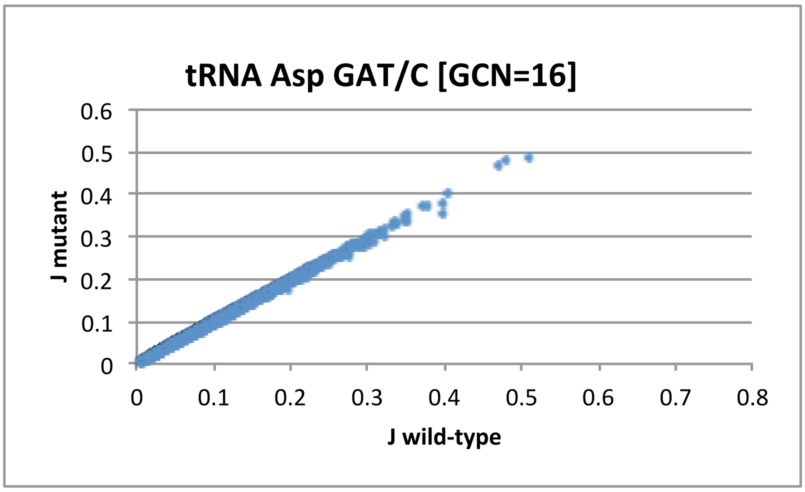
tRNA Asp GAT/C [GCN=16]
J mutant
0
0.1
0.2
0.3
0.4
0.5
0.6
0
0.1
0.2
0.3
0.4
0.5
0.6
0.7
0.8
J wild-type

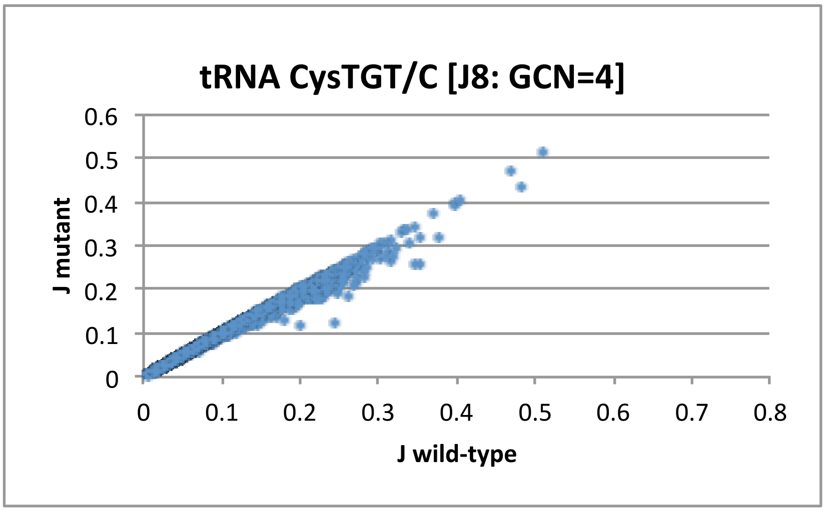
tRNA CysTGT/C [J8: GCN=4]
J mutant
J wild-type
0
0.1
0.2
0.3
0.4
0.5
0.6
0.7
0.8

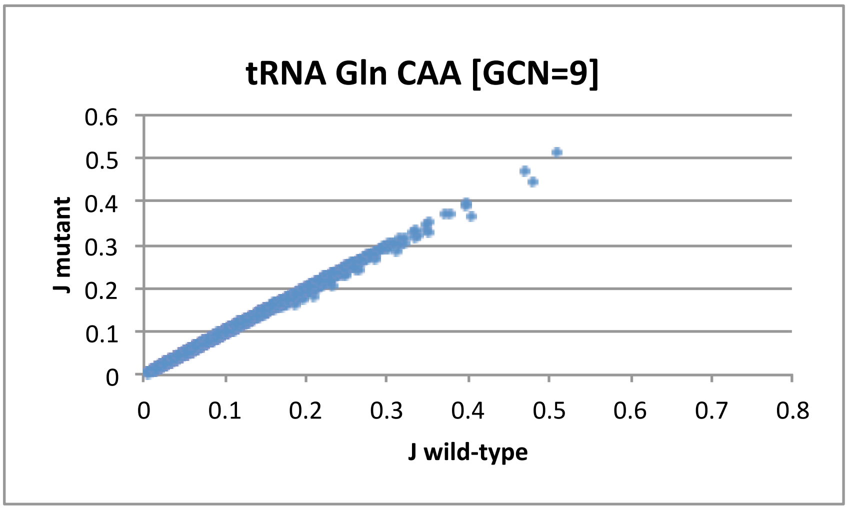
tRNA Gln CAA [GCN=9]
J mutant
J wild-type
0
0.1
0.2
0.3
0.4
0.5
0.6
0.7
0.8

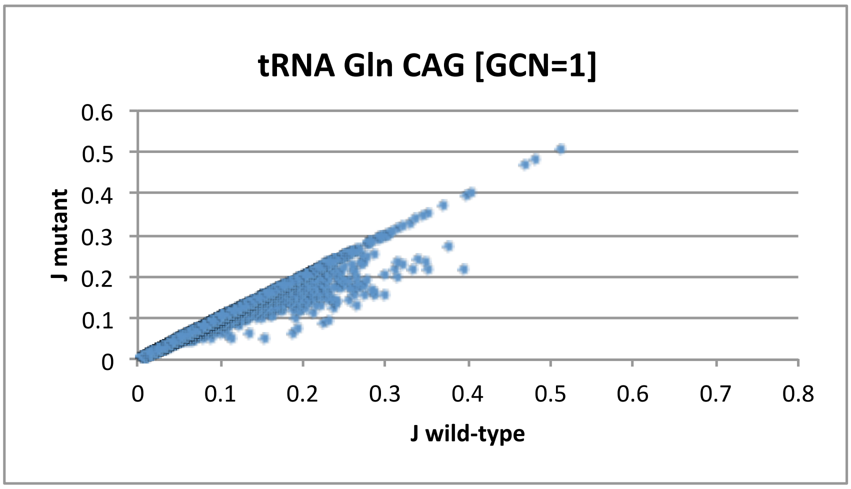
tRNA Gln CAG [GCN=1]
J mutant
J wild-type
0
0.1
0.2
0.3
0.4
0.5
0.6
0.7
0.8

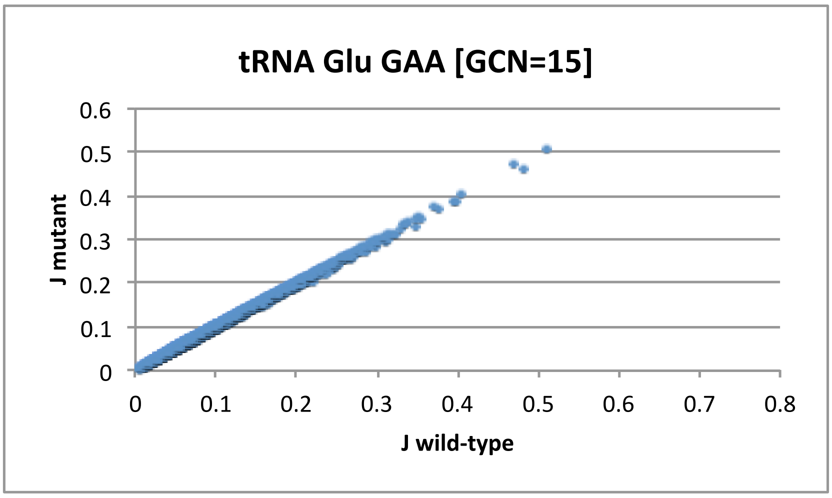
tRNA Glu GAA [GCN=15]
J mutant
0.6
0.5
0.4
0.3
0.2
0.1
0
0
0.1
0.2
0.3
0.4
0.5
0.6
0.7
0.8
J wild-type

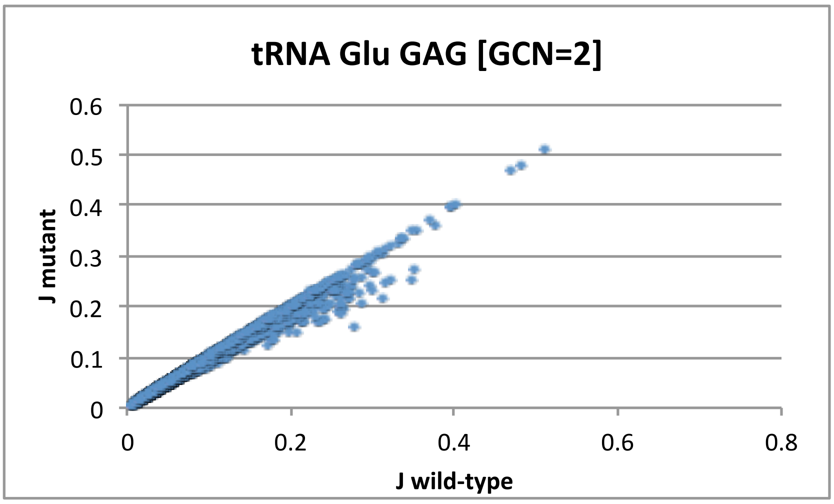
tRNA Glu GAG [GCN=2]
J mutant
0.6
0.5
0.4
0.3
0.2
0.1
0
0
0.2
0.4
0.6
0.8
J wild-type

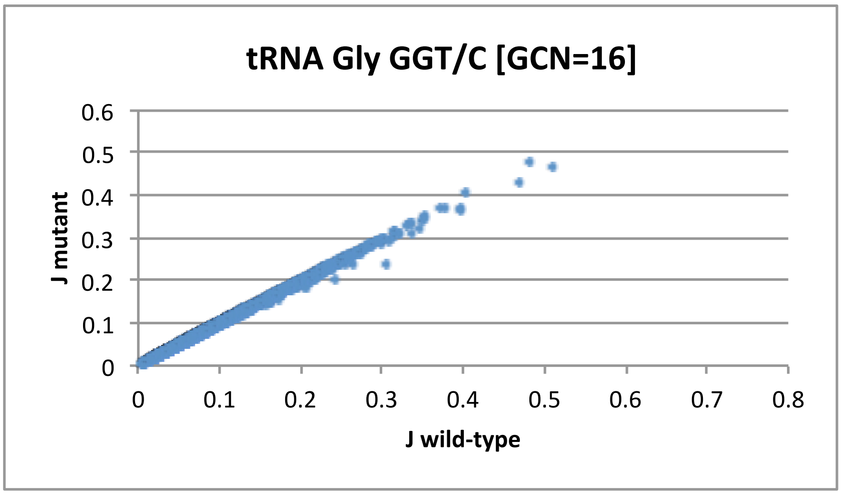
tRNA Gly GGT/C [GCN=16]
J mutant
0.6
0.5
0.4
0.3
0.2
0.1
0
0
0.1
0.2
0.3
0.4
0.5
0.6
0.7
0.8
J wild-type

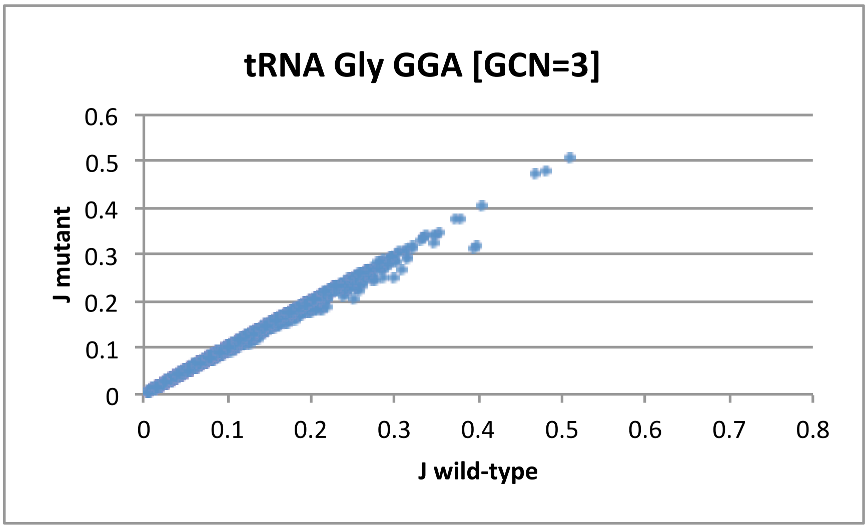
tRNA Gly GGA [GCN=3]
J mutant
0.6
0.5
0.4
0.3
0.2
0.1
0
0 0.1 0.2 0.3 0.4 0.5 0.6 0.7 0.8
J wild-type

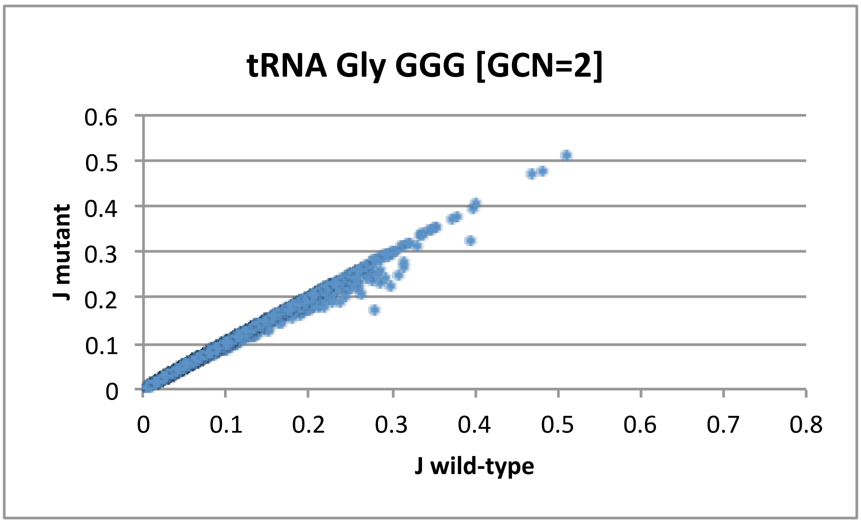
tRNA Gly GGG [GCN=2]
J mutant
0.6
0.5
0.4
0.3
0.2
0.1
0
0 0.1 0.2 0.3 0.4 0.5 0.6 0.7 0.8
J wild-type

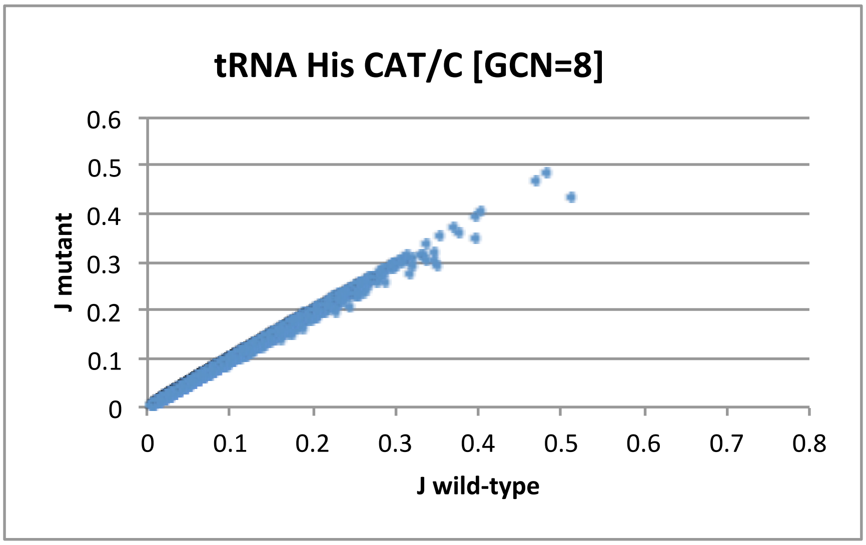
tRNA His CAT/C [GCN=8]
J mutant
0.6
0.5
0.4
0.3
0.2
0.1
0
0 0.1 0.2 0.3 0.4 0.5 0.6 0.7 0.8
J wild-type

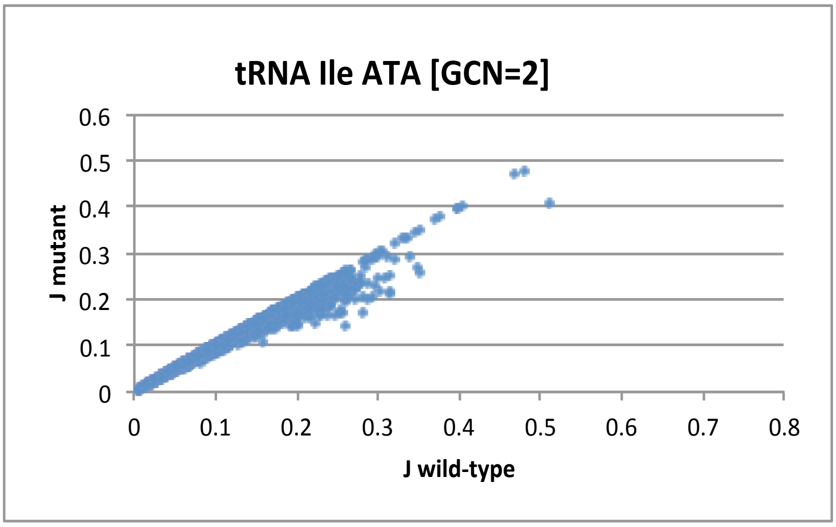
tRNA Ile ATA [GCN=2]
J mutant
J wild-type
0
0.1
0.2
0.3
0.4
0.5
0.6
0.7
0.8

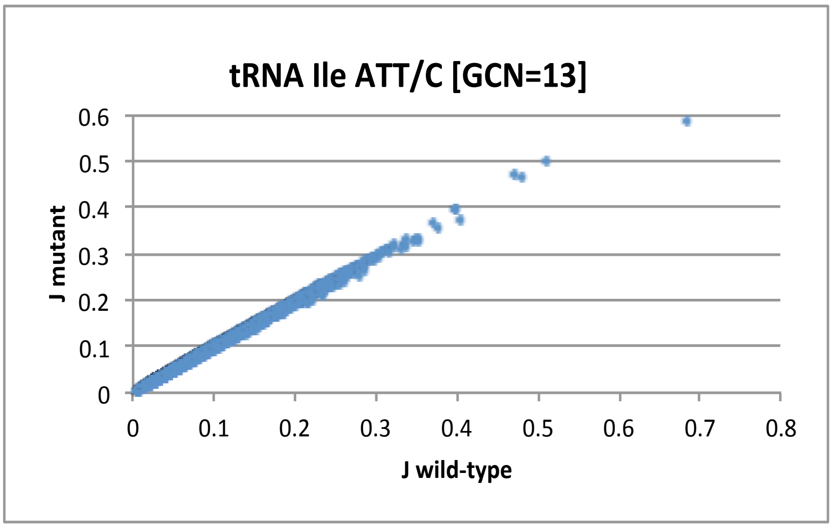
tRNA Ile ATT/C [GCN=13]
J mutant
J wild-type
0
0.1
0.2
0.3
0.4
0.5
0.6
0.7
0.8

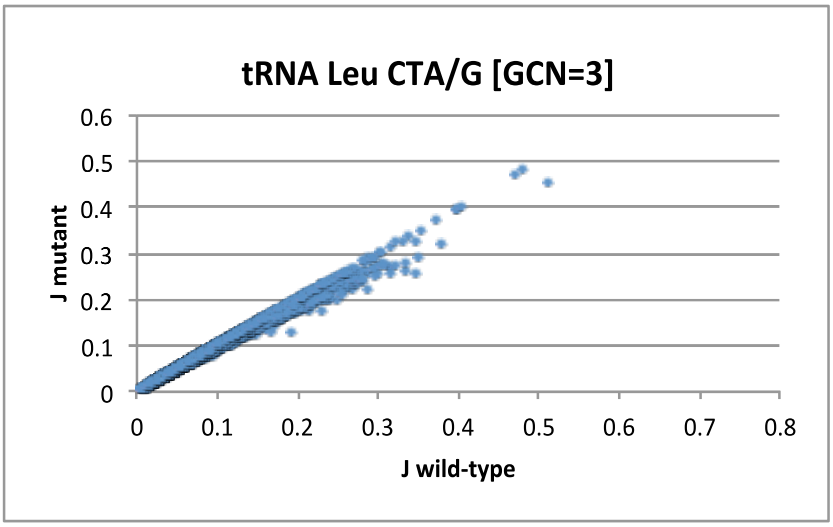
tRNA Leu CTA/G [GCN=3]
J mutant
J wild-type
0
0.1
0.2
0.3
0.4
0.5
0.6
0.7
0.8

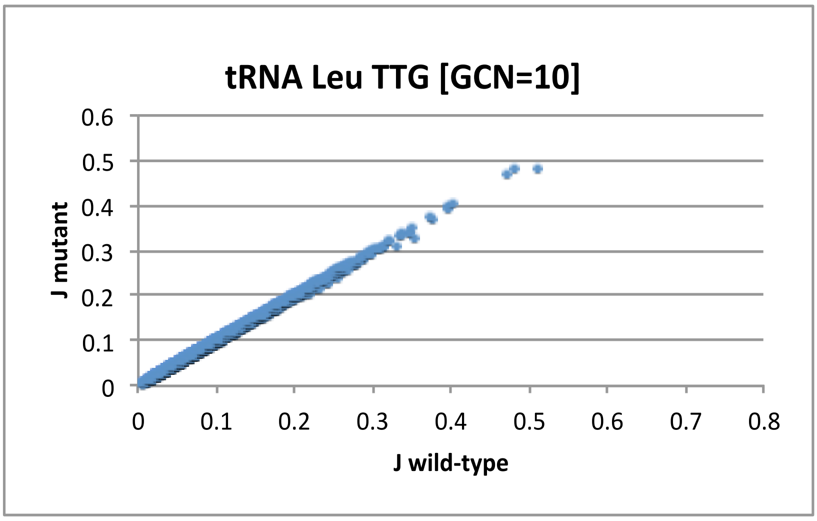
tRNA Leu TTG [GCN=10]
J mutant
J wild-type
0.6
0.5
0.4
0.3
0.2
0.1
0
0 0.1 0.2 0.3 0.4 0.5 0.6 0.7 0.8

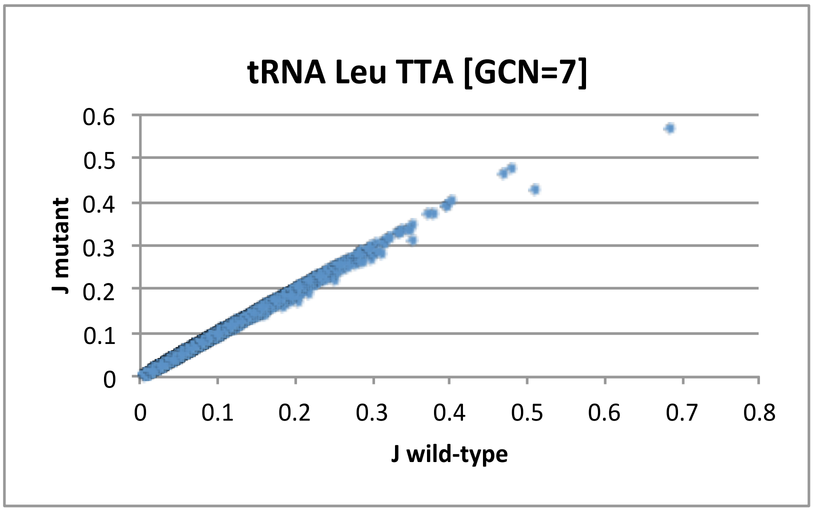
tRNA Leu TTA [GCN=7]
J mutant
J wild-type
0.6
0.5
0.4
0.3
0.2
0.1
0
0 0.1 0.2 0.3 0.4 0.5 0.6 0.7 0.8

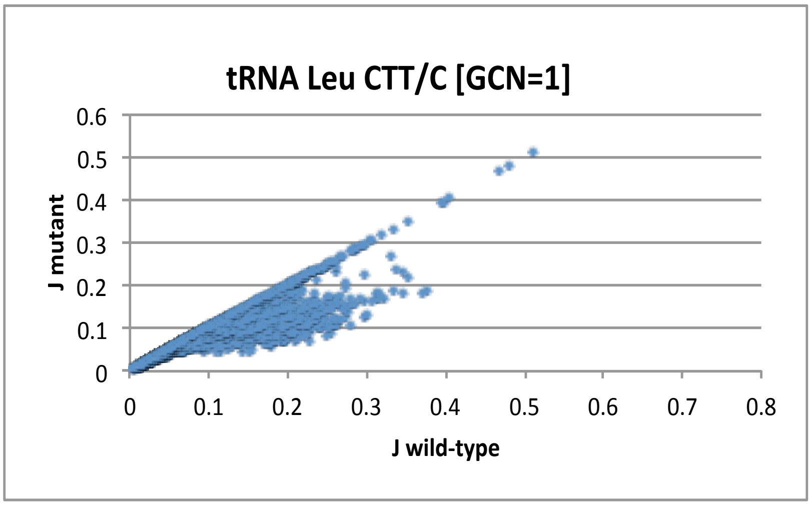
tRNA Leu CTT/C [GCN=1]
J mutant
J wild-type
0.6
0.5
0.4
0.3
0.2
0.1
0
0 0.1 0.2 0.3 0.4 0.5 0.6 0.7 0.8

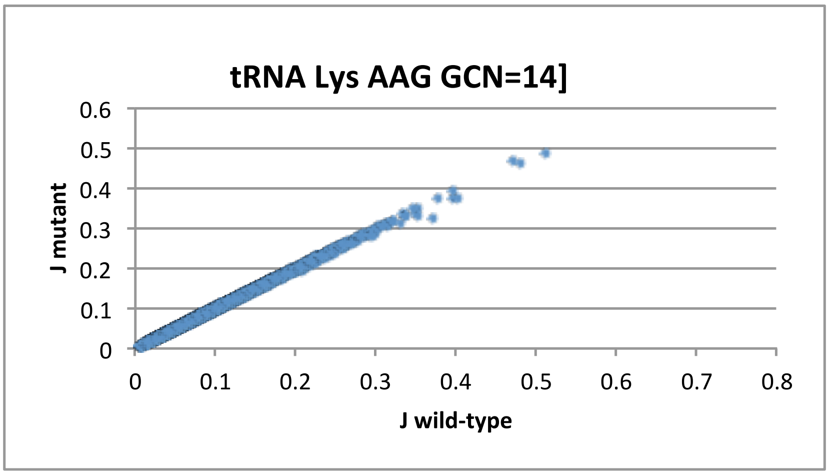
tRNA Lys AAG GCN=14]
0.6
0.5
0.4
0.3
0.2
0.1
0
J mutant
0
0.1
0.2
0.3
0.4
0.5
0.6
0.7
0.8
J wild-type

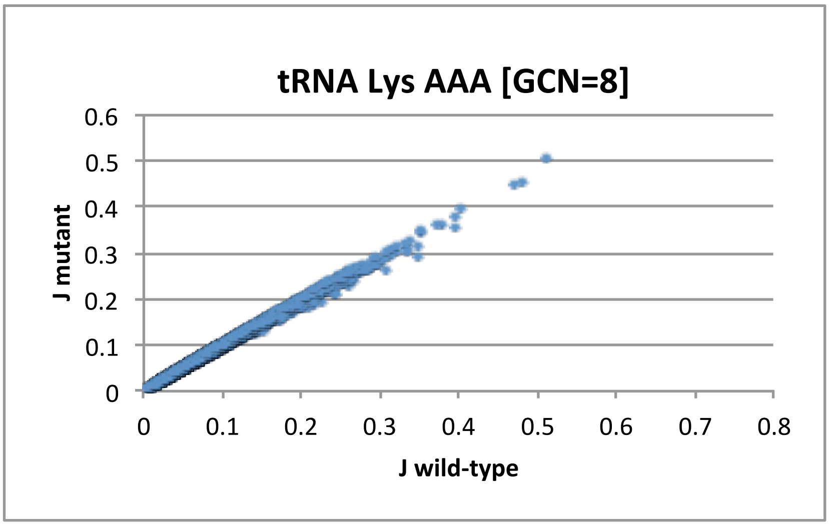
tRNA Lys AAA [GCN=8]
0.6
0.5
0.4
0.3
0.2
0.1
0
J mutant
0
0.1
0.2
0.3
0.4
0.5
0.6
0.7
0.8
J wild-type

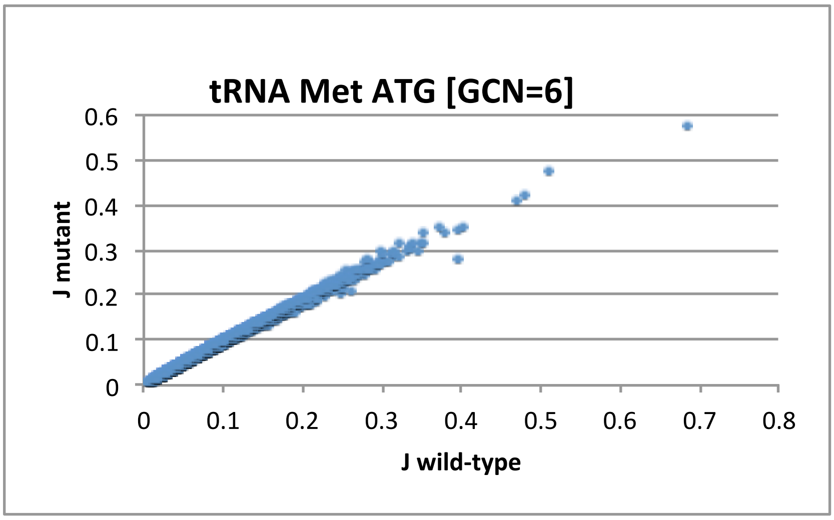
tRNA Met ATG [GCN=6]
0.6
0.5
0.4
0.3
0.2
0.1
0
J mutant
0
0.1
0.2
0.3
0.4
0.5
0.6
0.7
0.8
J wild-type

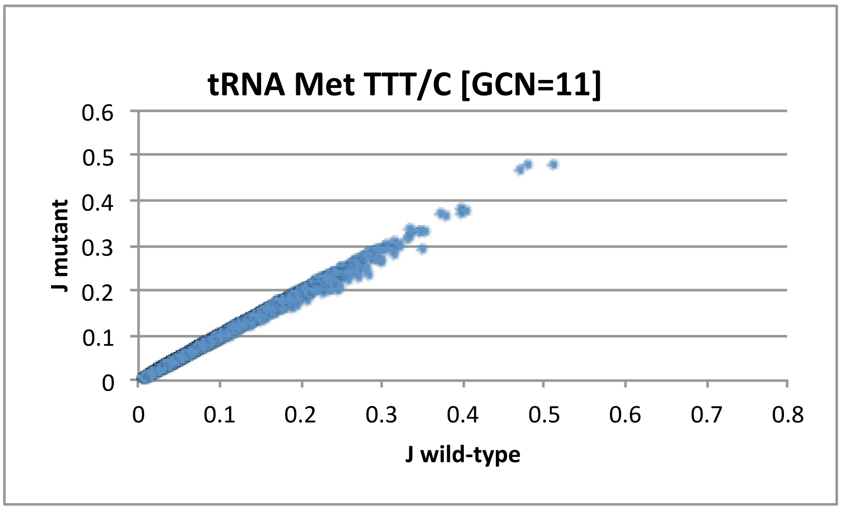
tRNA Met TTT/C [GCN=11]
J mutant
0.6
0.5
0.4
0.3
0.2
0.1
0
0
0.1
0.2
0.3
0.4
0.5
0.6
0.7
0.8
J wild-type

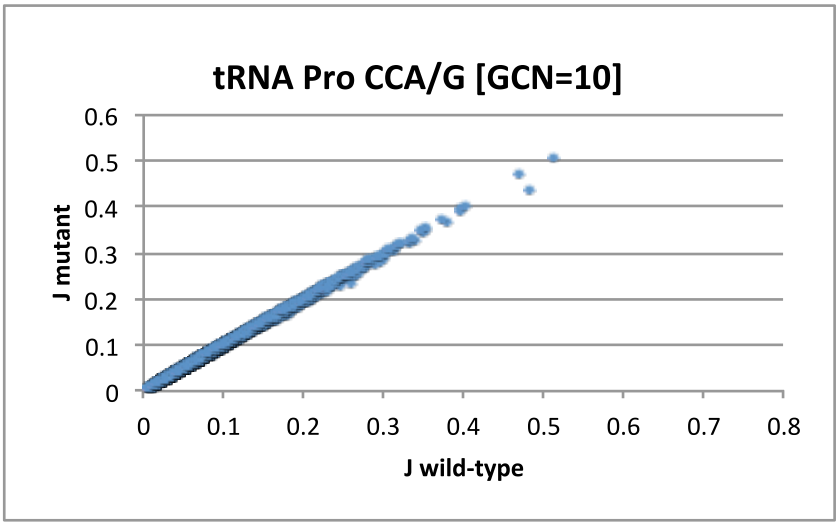
tRNA Pro CCA/G [GCN=10]
J mutant
0.6
0.5
0.4
0.3
0.2
0.1
0
0
0.1
0.2
0.3
0.4
0.5
0.6
0.7
0.8
J wild-type

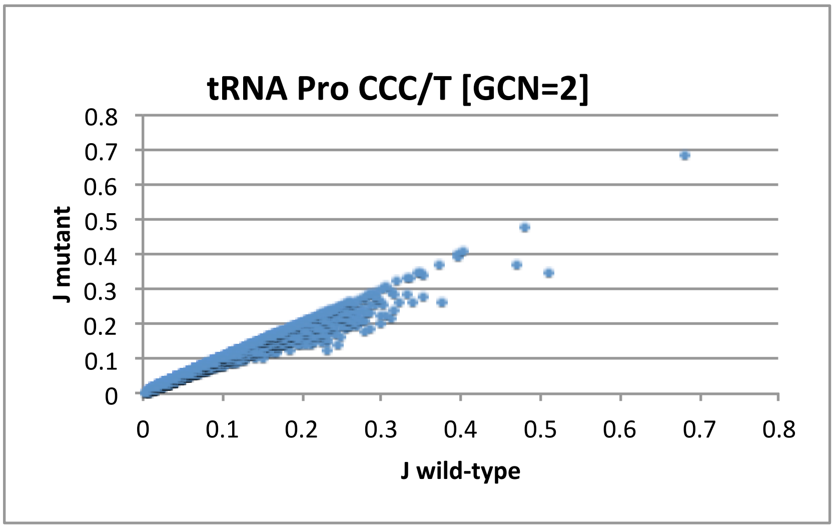
tRNA Pro CCC/T [GCN=2]
J mutant
0.8
0.7
0.6
0.5
0.4
0.3
0.2
0.1
0
0
0.1
0.2
0.3
0.4
0.5
0.6
0.7
0.8
J wild-type

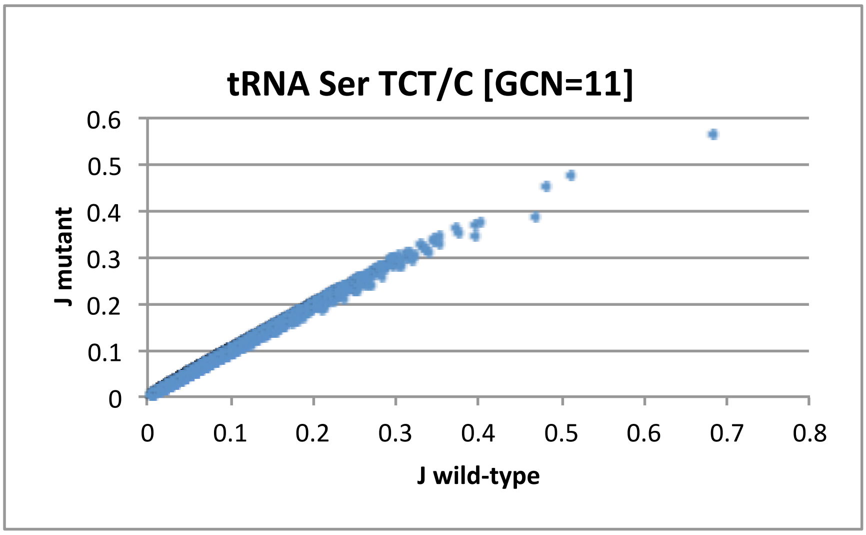
tRNA Ser TCT/C [GCN=11]
J mutant
J wild-type
0
0.1
0.2
0.3
0.4
0.5
0.6
0.7
0.8

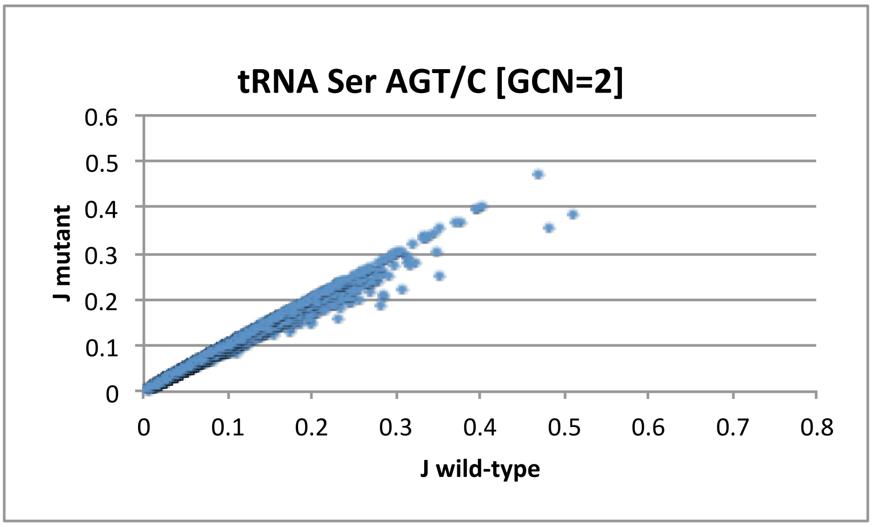
tRNA Ser AGT/C [GCN=2]
J mutant
J wild-type
0
0.1
0.2
0.3
0.4
0.5
0.6
0.7
0.8

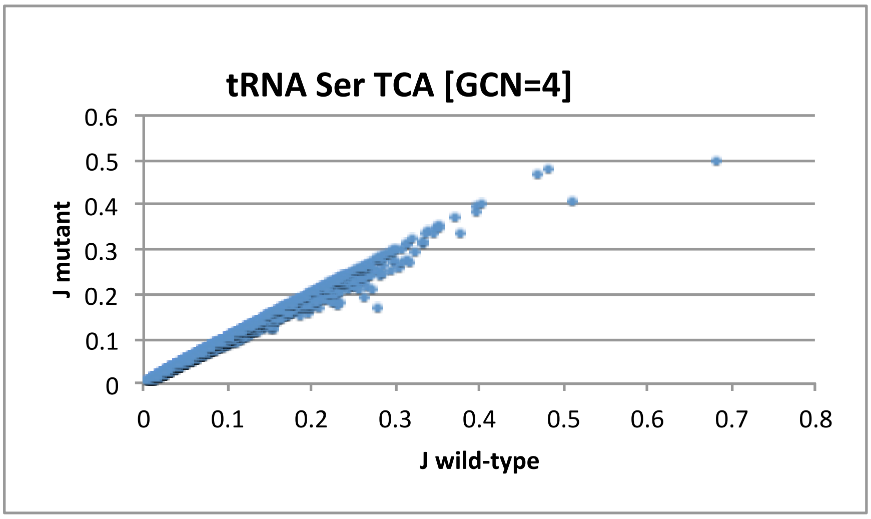
tRNA Ser TCA [GCN=4]
J mutant
J wild-type
0
0.1
0.2
0.3
0.4
0.5
0.6
0.7
0.8

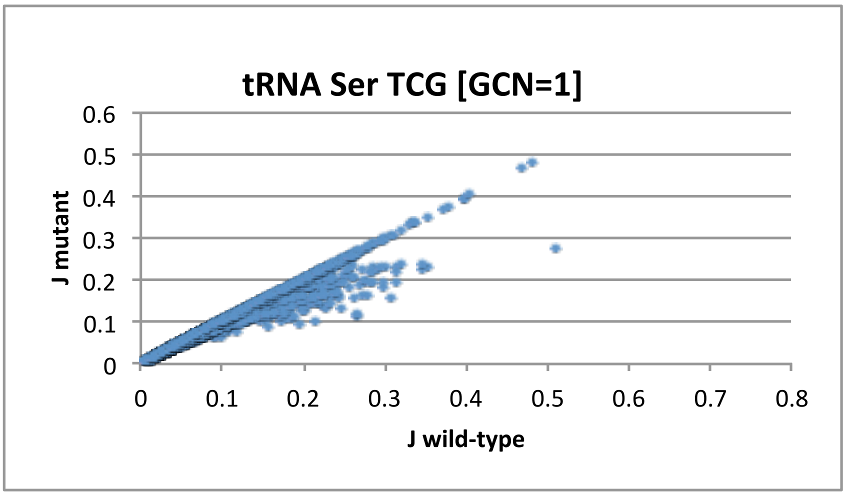
tRNA Ser TCG [GCN=1]
J mutant
J wild-type
0
0.1
0.2
0.3
0.4
0.5
0.6
0.7
0.8

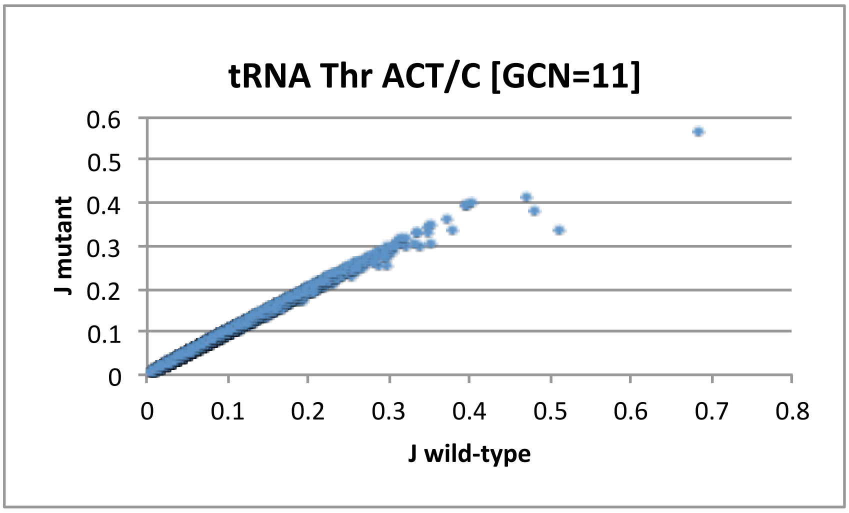
tRNA Thr ACT/C [GCN=11]
J mutant
J wild-type
0
0.1
0.2
0.3
0.4
0.5
0.6
0.7
0.8

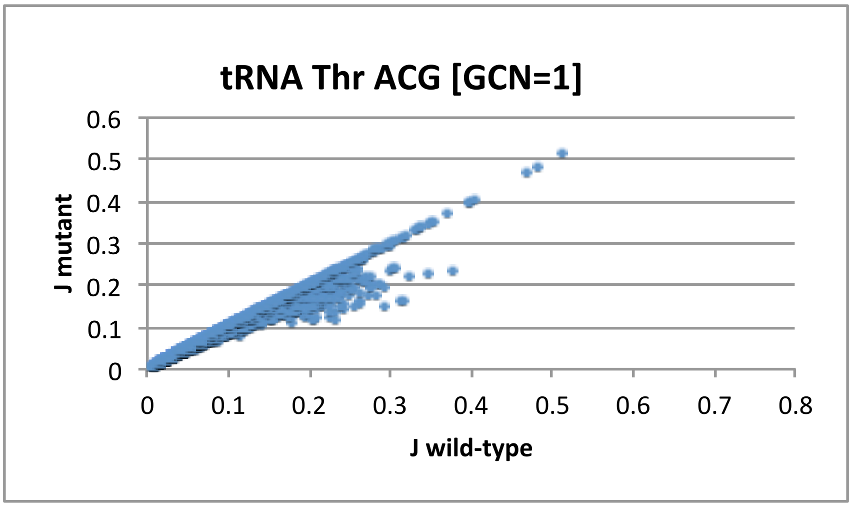
tRNA Thr ACG [GCN=1]
J mutant
J wild-type
0
0.1
0.2
0.3
0.4
0.5
0.6
0.7
0.8

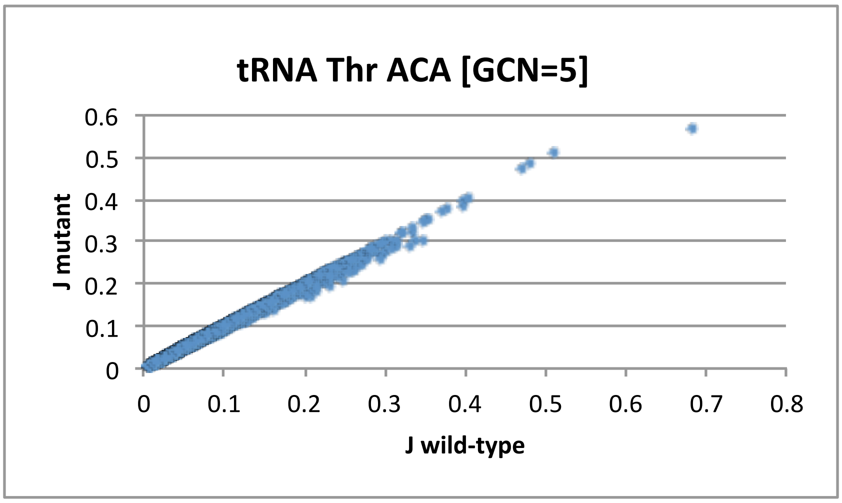
tRNA Thr ACA [GCN=5]
J mutant
J wild-type
0
0.1
0.2
0.3
0.4
0.5
0.6
0.7
0.8

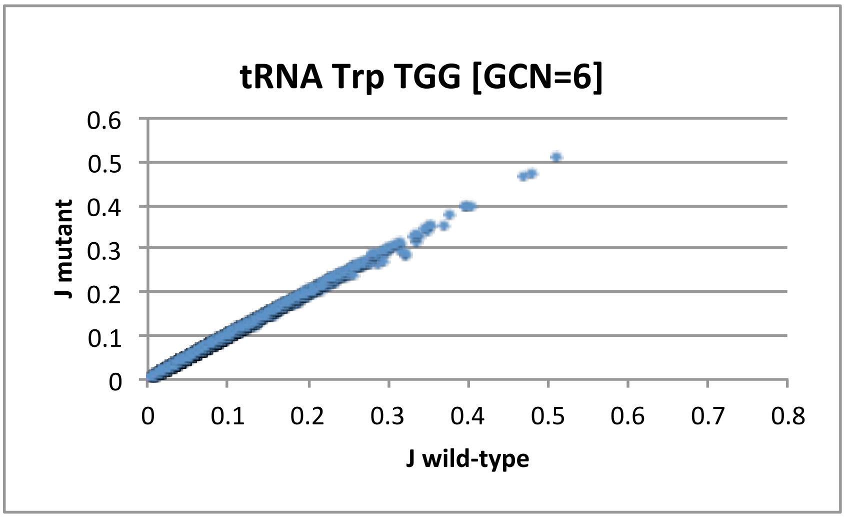
tRNA Trp TGG [GCN=6]
J mutant
J wild-type
0
0.1
0.2
0.3
0.4
0.5
0.6
0.7
0.8

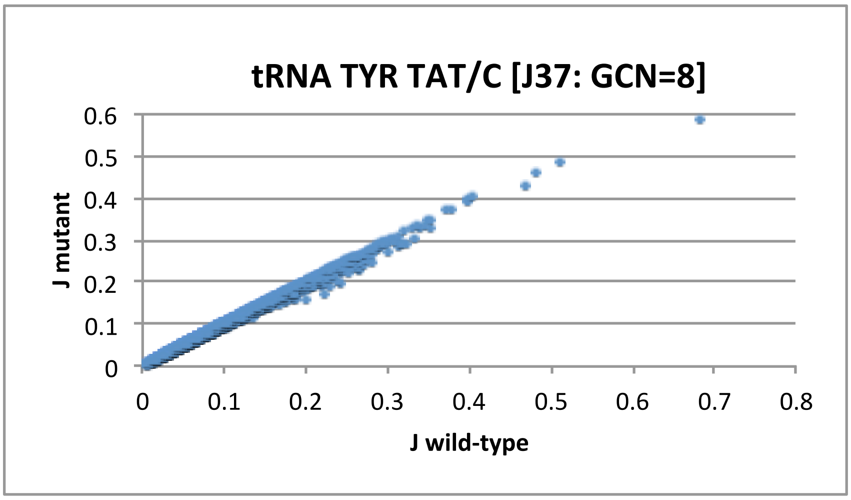
tRNA TYR TAT/C [J37: GCN=8]
J mutant
J wild-type
0
0.1
0.2
0.3
0.4
0.5
0.6
0.7
0.8

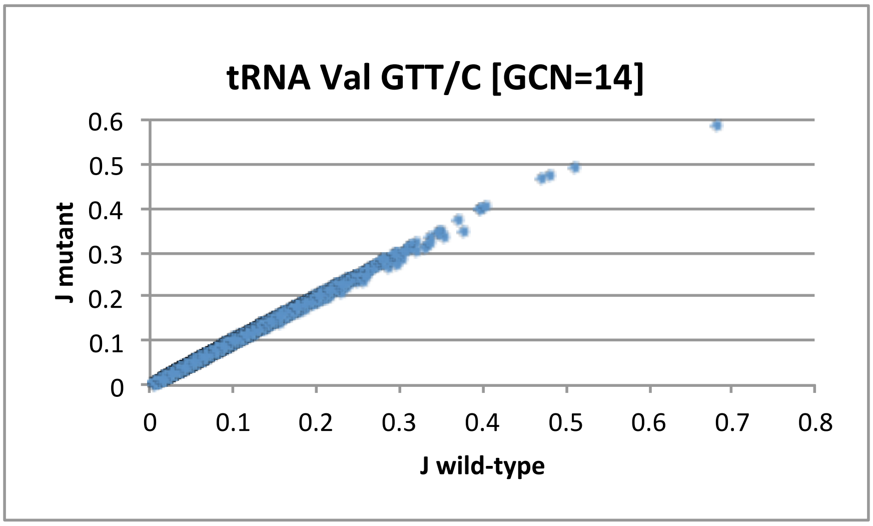
tRNA Val GTT/C [GCN=14]
J mutant
J wild-type
0
0.1
0.2
0.3
0.4
0.5
0.6
0.7
0.8

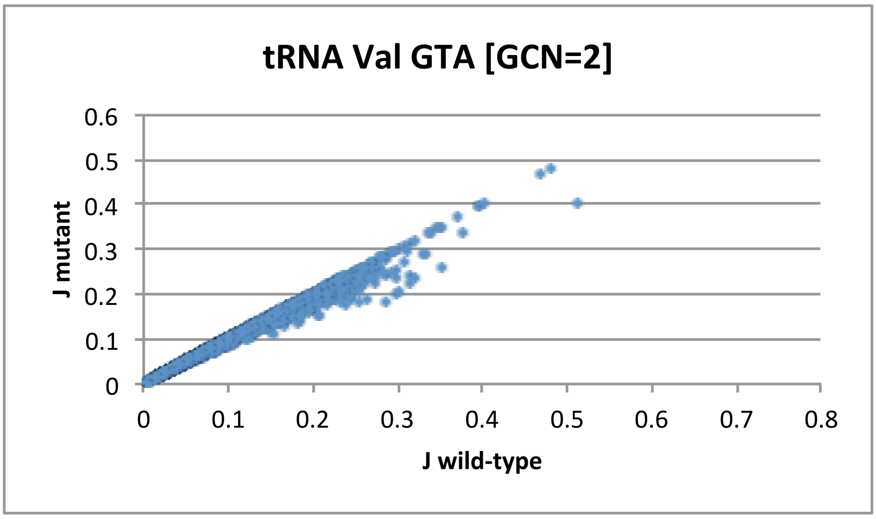
tRNA Val GTA [GCN=2]
J mutant
J wild-type
0
0.1
0.2
0.3
0.4
0.5
0.6
0.7
0.8

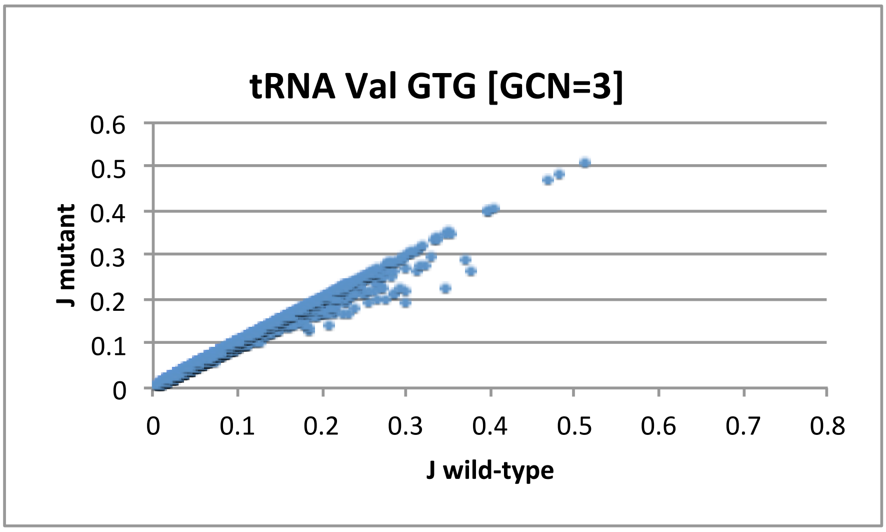
tRNA Val GTG [GCN=3]
J mutant
J wild-type
0
0.1
0.2
0.3
0.4
0.5
0.6
0.7
0.8
