# Supplementary material for: Identification of the mRNA targets of tRNA-specific regulation using genome-wide simulation of translation
Source: Nucleic Acids Res. 2016 Jul 12;44(19):9231–44. doi: 10.1093/nar/gkw630 (PMC5100601; doi:10.1093/nar/gkw630)
Supplement: SUPPLEMENTARY DATA [file supp_gkw630_nar-01103-n-2016-File011.docx]

**Table S1:** CAG-containing ORFs analysed

|  | **Gene name (systematic)** | **Gene name** | **Length (codons)** | **CAG codon content** | **Proportional CAG content (%)** | **Translation rate in mutant (J-mutant)** | **Translation rate in wild-type (J-wild-type)** | **Ratio J-mut/J-wild-type** |
| --- | --- | --- | --- | --- | --- | --- | --- | --- |
|  | YIL152W |  | 235 | 19 | 8.09% | 0.0517 | 0.1529 | 0.3379 |
|  | YFR008W | FAR7 | 221 | 12 | 5.43% | 0.0550 | 0.1112 | 0.4947 |
|  | YLR254C | NDL1 | 189 | 5 | 2.65% | 0.1389 | 0.2393 | 0.5805 |
| **TEST ORFS** | YJR086W | STE18 | 110 | 5 | 4.55% | 0.0786 | 0.1960 | 0.4010 |
|  | YDL012C |  | 107 | 12 | 11.22% | 0.0631 | 0.1355 | 0.4660 |
|  | YMR159C | ATG16 | 150 | 1 | 0.67% | 0.2339 | 0.3459 | 0.6761 |
|  | YML030W | RCF1 | 159 | 2 | 1.26% | 0.1931 | 0.2772 | 0.6966 |
|  | YLR104W | LCL2 | 131 | 7 | 5.34% | 0.0973 | 0.2296 | 0.4238 |
|  |  |  |  |  |  |  |  |  |
|  | YDR354W | TRP4 | 380 | 3 | 0.79% | 0.0791 | 0.0787 | 1.0054 |
|  | YOL086C | ADH1 | 348 | 0 | 0.00% | 0.2202 | 0.2191 | 1.0048 |
| **CONTROL** | YAL038W | CDC19 | 500 | 0 | 0.00% | 0.1704 | 0.1694 | 1.0059 |
| **ORFS** | YMR043W | MCM1 | 286 | 29 | 10.14% | 0.0745 | 0.0797 | 0.9350 |
|  | YHL020C | OPI1 | 404 | 30 | 7.43% | 0.0474 | 0.0460 | 1.0308 |
|  | YBR233W | PBP2 | 413 | 6 | 1.45% | 0.0740 | 0.0743 | 0.9953 |
